# Surface-guided deep-inspiration breath-hold radiotherapy for left-sided breast cancer: a systematic review and meta-analysis of dosimetric outcomes and image-verified setup accuracy

**DOI:** 10.3389/fonc.2026.1936561

**Published:** 2026-09-17

**Authors:** Xiaoyan Wu, Huaqiong Wang, Peng Wu, Yin Zhang

**Affiliations:** 1 National Cancer Center/National Clinical Research Center for Cancer/Cancer Hospital & Shenzhen Hospital, Chinese Academy of Medical Sciences and Peking Union Medical College, Shenzhen, China; 2 West China School of Medicine, Sichuan University, Chengdu, China; 3 Huizhou University, Huizhou, Guangdong, China

**Keywords:** breast cancer, deep-inspiration breath-hold, dosimetry, meta-analysis, optical surface monitoring, setup accuracy, surface-guided radiotherapy

## Abstract

**Background:**

Deep-inspiration breath-hold (DIBH) reduces cardiac and pulmonary radiation exposure during radiotherapy for left-sided breast cancer. Surface-guided radiotherapy (SGRT) and optical surface monitoring systems (OSMS) can support patient positioning and breath-hold monitoring, but evidence specific to SGRT/OSMS-assisted DIBH workflows remains fragmented.

**Methods:**

PubMed, Web of Science, Embase, and Cochrane CENTRAL were searched from inception to May 21, 2026. Studies using SGRT/OSMS-assisted DIBH and reporting extractable dosimetric or image-verified setup outcomes were eligible. Effects were expressed as mean differences (MDs) with 95% confidence intervals (CIs); dosimetric MDs were defined as SGRT/OSMS-assisted DIBH minus FB, with negative values indicating lower doses. Random-effects meta-analyses were used for dosimetric and comparative setup outcomes, whereas single-arm setup data were pooled separately.

**Results:**

A total of 52 studies were included. Compared with free breathing (FB), SGRT/OSMS-assisted DIBH was associated with lower mean heart dose (MD = -1.21 Gy, 95% CI -1.48 to -0.93), mean left anterior descending coronary artery (LAD) dose (MD = -7.05 Gy, 95% CI -9.39 to -4.71), ipsilateral (left) lung mean dose (MD = -0.88 Gy, 95% CI -1.34 to -0.42), and ipsilateral lung V20 (MD = -2.30 percentage points, 95% CI -3.27 to -1.32). Comparative meta-analyses found no statistically significant pooled between-group differences in image-verified 3D-vector, left-right/lateral (LR/LAT), superior-inferior/longitudinal/craniocaudal (SI/LNG/CC), or anterior-posterior/vertical (AP/VRT) residual setup errors; this finding does not establish equivalence or non-inferiority. Sensitivity analyses generally supported the main conclusions, although the magnitude of the ipsilateral lung V20 effect was sensitive to the statistical model.

**Conclusions:**

SGRT/OSMS-assisted DIBH was associated with lower cardiac, LAD, and ipsilateral lung doses in left-sided breast radiotherapy. Comparative setup analyses found no statistically significant pooled differences from the comparator workflows reported in the primary studies; this does not establish equivalence or non-inferiority. Current evidence supports SGRT/OSMS for external positioning, real-time DIBH monitoring, and quality assurance, while appropriate internal image verification should be retained according to target complexity, the SGRT system, and the local quality assurance program.

**Systematic review registration:**

https://www.crd.york.ac.uk/PROSPERO/view/CRD420261439818, identifier CRD420261439818.

## Introduction

Adjuvant radiotherapy is an integral component of curative treatment after breast-conserving surgery or mastectomy and improves local control and long-term breast cancer outcomes. In left-sided breast irradiation, however, the heart, left anterior descending coronary artery (LAD), and ipsilateral lung may lie close to the target and tangential fields, making reduction of unnecessary organ-at-risk exposure clinically important. The risk of major coronary events increases with increasing mean heart dose, with no clear safe threshold, and lung irradiation may also increase the risk of radiation-related pulmonary toxicity (1–5).

DIBH increases lung volume, lowers the diaphragm, and moves the heart away from the chest wall and tangential treatment fields, thereby reducing organ-at-risk exposure. Previous meta-analyses have shown that DIBH reduces cardiac, LAD, and ipsilateral lung doses compared with free breathing (FB), and recent work has specifically evaluated DIBH in volumetric modulated arc therapy (6–10). DIBH, however, is not a single implementation strategy: active breathing control, respiratory gating, visual feedback, spirometry, and surface guidance systems differ in training, monitoring, gating thresholds, and image verification. Accordingly, general DIBH-versus-FB evidence cannot, by itself, characterize the performance of a specific monitoring workflow.

SGRT/OSMS uses optical surface imaging to support daily positioning, breath-hold coaching, real-time motion monitoring, and, in some systems, beam gating without additional ionizing imaging dose. Breast DIBH is an important clinical application of SGRT. Nevertheless, the external surface is a surrogate for internal anatomy; locally validated action thresholds, quality assurance, and appropriate internal image verification remain necessary when the position of the heart, LAD, chest wall, or regional nodal targets must be confirmed (11–13). Previous systematic reviews have mainly compared SGRT with tattoo- or laser-based breast positioning in free-breathing settings and have not jointly synthesized SGRT-DIBH dosimetry and image-verified residual setup accuracy as two distinct outcome domains (14).

We therefore conducted a systematic review and meta-analysis focused on SGRT/OSMS-assisted DIBH for left-sided breast radiotherapy. The prespecified questions were whether this workflow was associated with lower cardiac and pulmonary doses than FB and whether image-verified residual setup outcomes supported reproducible treatment delivery. Dosimetric and setup accuracy evidence were analyzed separately because they address different clinical questions; setup data do not directly establish organ dose or clinical outcomes.

## Methods

### Protocol and reporting

This systematic review and meta-analysis followed the PRISMA 2020 statement (15) and was registered in PROSPERO (CRD420261439818). The protocol prespecified eligibility criteria, outcome domains, data handling, and statistical analysis. Only published and publicly available data were used; therefore, additional ethics approval was not required.

### Search strategy

PubMed, Web of Science, Embase, and Cochrane CENTRAL were searched from database inception through May 21, 2026, with the final update completed on that date. The strategy combined controlled vocabulary and free-text terms for left-sided breast cancer, radiotherapy, DIBH, surface guidance/OSMS, dosimetry, image verification, and setup accuracy, with syntax adapted to each database. Before deduplication, 3,815 records were identified: 913 from PubMed, 1,302 from Web of Science, 286 from Embase, and 1,314 from CENTRAL. Complete line-by-line search histories for each database are provided in **Supplementary Tables S1.1–S1.4**.

### Eligibility criteria and outcomes

Eligible reports included patients receiving radiotherapy to the left breast or chest wall, with or without regional nodal irradiation or a boost, and used an SGRT/OSMS-assisted DIBH workflow. Comparative dosimetric planning or clinical studies were required to report both an SGRT/OSMS-assisted DIBH condition and an FB condition. Image-verified setup studies were required to report residual error data under SGRT/OSMS-assisted DIBH; comparative and single-arm data were synthesized separately. Reviews, meta-analyses, case reports, conference abstracts, duplicate or overlapping cohorts, studies with unclear DIBH technology, and reports without extractable quantitative data were excluded. For overlapping populations, the report with the most complete or most relevant outcome data was retained.

Primary dosimetric outcomes were heart Dmean, LAD Dmean, ipsilateral lung Dmean, and ipsilateral lung V20. Because only left-sided breast cancer was included, the ipsilateral lung refers to the left lung throughout this article. Other cardiac, LAD, and lung dose volume parameters were treated as secondary outcomes. For setup accuracy, absolute residual errors and signed directional displacements were extracted as distinct metrics: absolute values describe error magnitude, whereas signed values describe displacement direction and were not interpreted as error magnitude. Directional labels were harmonized as left-right/lateral (LR/LAT), superior–inferior/longitudinal/craniocaudal (SI/LNG/CC), and anterior–posterior/vertical (AP/VRT).

### Data collection and outcome definitions

Two reviewers independently screened titles and abstracts, assessed full texts, extracted data, and evaluated methodological quality; disagreements were resolved by discussion. The extracted information included study design, patient and treatment characteristics, target extent and treatment technique, SGRT/OSMS system, reference surface and region of interest (ROI), breath-hold workflow, gating/action thresholds, comparator, imaging-verification modality, timing of error measurement, imaging verification frequency/number of fractions, outcome definitions, sample size, means, and measures of dispersion. Image-verified setup error was defined as the geometric positioning discrepancy identified by image verification relative to the planned or image-based reference position after SGRT/OSMS-guided surface positioning. Because image verification and correction workflows were not fully uniform across the included studies, this outcome was extracted according to the timing and definition reported in each primary study. Imaging modality, error measurement timing, and verification frequency/number of fractions were also recorded; these details are provided in **Supplementary Appendix 9, Supplementary Table S9.1**. Information not reported in the primary study was coded as NR.

Dosimetric outcomes were harmonized using the following rules. Heart Dmean was extracted as the mean dose to the heart/whole-heart contour reported by the source study. LAD Dmean was extracted as the mean dose to an explicitly delineated LAD contour, with the reported RTOG, ESTRO, Feng, other contouring atlas, or segmentation range recorded. Ipsilateral lung Dmean and V20 were included only when the left/ipsilateral lung was explicitly used as the calculation volume. Dmean and Dmax were standardized to gray, Vx to percentages, between-group Vx mean differences to percentage points, and setup errors to millimeters. Outcomes explicitly based on whole/bilateral-lung V20 or other anatomically non-equivalent structures were not pooled with the corresponding primary outcome; unreported contouring standards were coded as NR. When dispersion was reported as a CI, SE, IQR, range, or another convertible measure, it was converted using prespecified methods (16); data sources and conversion procedures are detailed in **Supplementary Appendix 3**.

### Methodological quality assessment

Because the evidence base consisted mainly of non-randomized dosimetric comparative studies and image-verification studies, some items of the conventional Newcastle–Ottawa Scale (NOS) did not fully capture the paired planning and image verification features of these designs. We therefore contextually adapted the decision criteria within the existing NOS framework (17). The adapted tool retained nine items: four selection domains, two comparability domains, and three outcome/reporting domains. Each item contributed one point for a maximum score of 9; scores of 7–9, 4–6, and 0–3 were classified as higher, moderate, and lower methodological quality, respectively. Scores were used to describe methodological quality and define the higher-quality-study sensitivity analysis, but not to weight pooled effects. Full decision rules and study-level ratings are provided in **Supplementary Appendix 4**.

### Statistical analysis

Study-level comparisons were pooled using restricted maximum likelihood (REML) random-effects models (18, 19), with effects expressed as MDs and 95% CIs. Dosimetric MDs were defined as SGRT/OSMS-assisted DIBH minus FB, so negative values indicate lower doses with SGRT/OSMS-assisted DIBH. Comparative setup analyses retained the group order reported in the primary studies and were interpreted only in that context; single-arm residual errors were summarized as pooled means. Between-study heterogeneity was assessed using *I*², *τ*², and Cochran’s Q, and 95% prediction intervals were reported when estimable. Prespecified sensitivity analyses for the primary dosimetric outcomes included a comparison of common-effect and random-effects models, exclusion of small studies, restriction to higher-quality studies, and leave-one-out analyses. When at least 10 study estimates contributed to an outcome, funnel plots and formal tests of asymmetry were evaluated. Pooled effects and 95% CIs were reported to two decimal places and *I*² to one decimal place. SGRT-system-stratified analyses were exploratory and were reported only when each SGRT-system subgroup contained at least three comparable independent studies.

## Results

### Study selection and characteristics

The search identified 3,815 records. After the removal of 1,661 duplicates, 2,154 records underwent title and abstract screening. Full-text retrieval was sought for 256 reports; two could not be retrieved, leaving 254 reports for full-text eligibility assessment, of which 202 were excluded. A total of 52 studies (20–71) were ultimately included in the systematic review and quantitative synthesis (**Figure 1**). The evidence base comprised comparative dosimetric planning/treatment studies, comparative image-verified setup studies, and single-arm residual error studies. The included studies represented multiple SGRT/OSMS systems and technical configurations, including AlignRT/VisionRT, C-RAD Sentinel/Catalyst, OSMS, Varian Identify, ExacTrac Dynamic, and other optical surface-tracking systems. Studies differed substantially in reference surfaces, ROIs, gating/action thresholds, visual feedback, image verification, and quality assurance workflows; detailed patient, treatment, SGRT system, and verification characteristics are provided in **Supplementary Appendices 2, 9**. To directly address technical heterogeneity across SGRT systems, we additionally performed exploratory SGRT-system-stratified analyses for primary dosimetric outcomes meeting the prespecified minimum study count; the results are presented in **Supplementary Appendix 9**.

**Figure 1:**
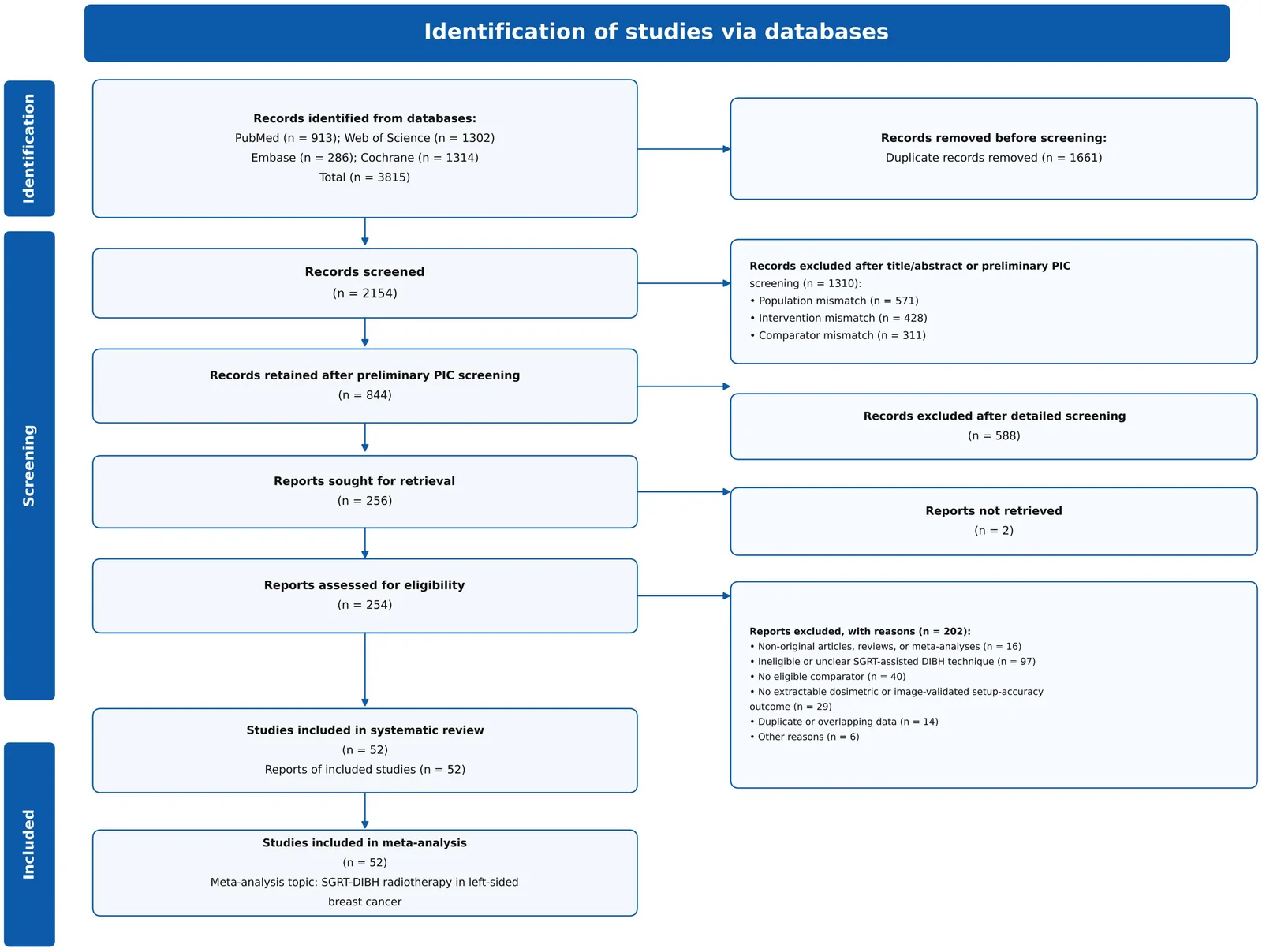
PRISMA 2020 flow diagram of study identification, screening, and inclusion. PIC denotes the population, intervention, and comparator framework used for preliminary screening.

### Methodological quality

On the adapted NOS-based assessment, six studies scored 9, 17 scored 8, 21 scored 7, and 8 scored 6. Accordingly, 44 studies were classified as higher methodological quality and eight as moderate quality; none was classified as lower quality. The most frequent limitations involved control of key comparability factors, the paired/planning control context, and completeness of setup verification reporting (**Supplementary Appendix 4**).

### Primary dosimetric outcomes

Compared with FB, SGRT/OSMS-assisted DIBH was associated with lower values for all four primary dosimetric outcomes: heart Dmean (*k* = 18, MD = -1.21 Gy, 95% CI -1.48 to -0.93; *I*² = 84.5%), LAD Dmean (*k* = 10, MD = -7.05 Gy, 95% CI -9.39 to -4.71; *I*² = 93.8%), ipsilateral lung Dmean (*k* = 12, MD = -0.88 Gy, 95% CI -1.34 to -0.42; *I*² = 72.2%), and ipsilateral lung V20 (*k* = 12, MD = -2.30 percentage points, 95% CI -3.27 to -1.32; *I*² = 93.6%) (**Figure 2**). Between-study heterogeneity was moderate to high for all four primary outcomes; effect magnitudes should therefore be interpreted together with prediction intervals and sensitivity analyses.

**Figure 2:**
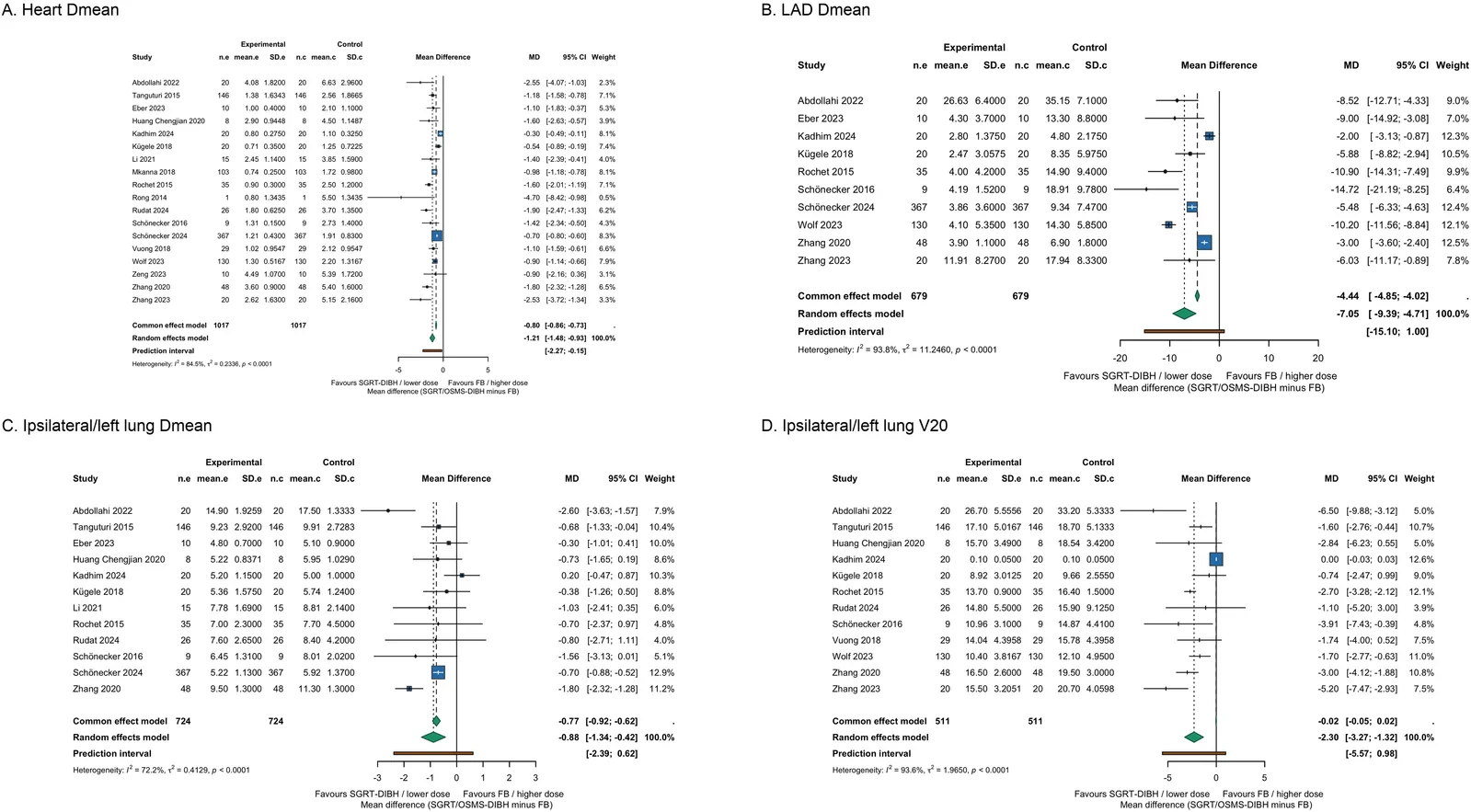
Forest plots of the primary dosimetric outcomes. **(A)** Heart Dmean; **(B)** LAD Dmean; **(C)** ipsilateral lung Dmean; and **(D)** ipsilateral lung V20. MD is defined as SGRT/OSMS-DIBH minus FB; negative values indicate lower doses with SGRT/OSMS-assisted DIBH. SGRT, surface-guided radiotherapy; OSMS, optical surface monitoring system; DIBH, deep-inspiration breath-hold; FB, free breathing; LAD, left anterior descending coronary artery; MD, mean difference.

### Secondary dosimetric outcomes

Secondary dosimetric outcomes were directionally consistent with the primary analyses. SGRT/OSMS-assisted DIBH was associated with lower heart Dmax (MD = -14.86 Gy, 95% CI -20.75 to -8.97), heart V5 (MD = -5.74 percentage points, 95% CI -7.20 to -4.28), heart V10 (MD = -3.75 percentage points, 95% CI -4.79 to -2.71), heart V20 (MD = -1.66 percentage points, 95% CI -1.91 to -1.40), and heart V30 (MD = -1.34 percentage points, 95% CI -2.07 to -0.61). LAD Dmax was also lower (MD = -20.11 Gy, 95% CI -27.30 to -12.93), as was ipsilateral lung V5 (MD = -2.67 percentage points, 95% CI -5.23 to -0.11). Several outcomes showed substantial heterogeneity; the complete pooled results and forest plots are provided in **Supplementary Appendices 5, 6**.

### Image-verified setup accuracy

The comparative meta-analyses found no statistically significant pooled between-group differences in 3D-vector, LR/LAT, SI/LNG/CC, or AP/VRT residual setup errors (**Figure 3**). These analyses were not equivalence or non-inferiority analyses; therefore, statistical non-significance should not be interpreted as evidence that the workflows are equivalent.

**Figure 3:**
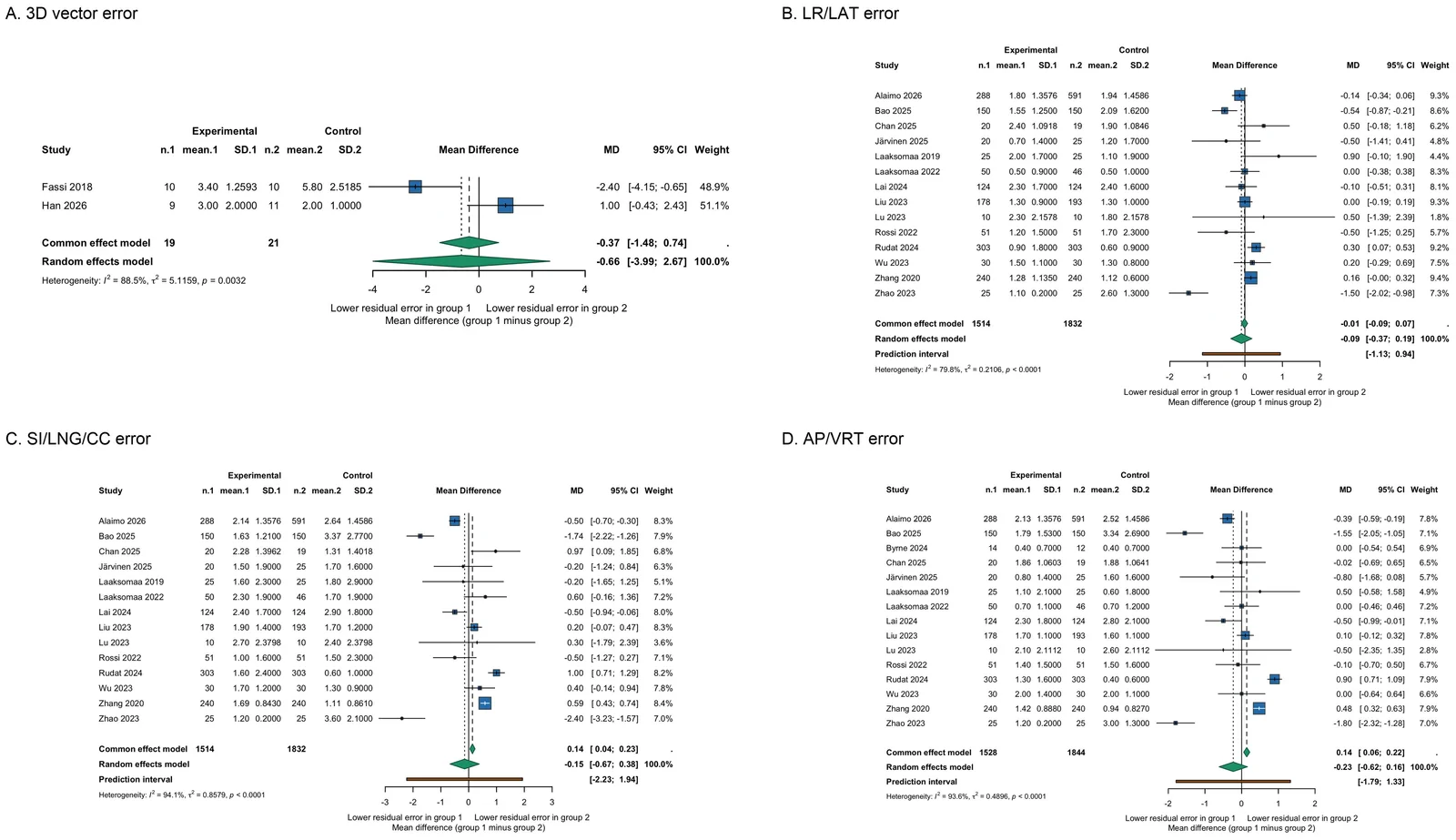
Forest plots of image-verified comparative setup accuracy. **(A)** 3D-vector error; **(B)** LR/LAT error; **(C)** SI/LNG/CC error; and **(D)** AP/VRT error. Effect direction follows the group order reported in the included studies and does not represent a uniform direction of benefit. LR/LAT, left-right/lateral; SI/LNG/CC, superior-inferior/longitudinal/craniocaudal; AP/VRT, anterior-posterior/vertical.

In single-arm analyses, pooled residual errors were 0.88 mm (95% CI 0.47 to 1.29) for LR/LAT, 2.02 mm (95% CI 1.40 to 2.64) for SI/LNG/CC, and 1.13 mm (95% CI 0.82 to 1.44) for AP/VRT. The pooled 3D-vector mean was 6.21 mm (95% CI 4.19 to 8.22), based on only two studies, with *I*² = 97.0%. The 3D vector is the magnitude of displacement combined across multiple orthogonal directions and is therefore defined differently from a single-axis error; accordingly, its numerical value is generally larger than an error measured along any one axis. The complete comparative and single-arm results are provided in **Supplementary Appendices 7, 8**.

### Robustness, heterogeneity, and small-study effects

The sensitivity analyses were generally consistent for heart Dmean, LAD Dmean, and ipsilateral lung Dmean. For ipsilateral lung V20, the common-effect estimate was close to the null (MD = -0.02 percentage points, 95% CI -0.05 to 0.02), whereas the random-effects and other sensitivity estimates retained negative point estimates; this outcome should therefore be interpreted cautiously. Small-study-effect signals were detected for heart Dmean and ipsilateral lung V20 (**Supplementary Appendix 5**). The exploratory SGRT-system-stratified analyses showed dose reductions in all analyzable system subgroups. For heart Dmean, the pooled estimates were AlignRT/VisionRT (*k* = 3, MD = -1.00 Gy, 95% CI -1.19 to -0.81), Catalyst/C-RAD (*k* = 5, MD = -1.04 Gy, 95% CI -1.57 to -0.51), OSMS (*k* = 4, MD = -1.72 Gy, 95% CI -2.02 to -1.43), and other SGRT/OSMS systems (*k* = 6, MD = -1.02 Gy, 95% CI -1.47 to -0.57). For ipsilateral lung V20, the estimates were Catalyst/C-RAD (*k* = 3, MD = -3.46 percentage points, 95% CI -6.90 to -0.02), OSMS (*k* = 4, MD = -2.98 percentage points, 95% CI -3.64 to -2.32), and other SGRT/OSMS systems (*k* = 5, MD = -1.06 percentage points, 95% CI -2.03 to -0.08). The magnitude of reduction varied: the largest heart Dmean reduction was observed in the OSMS subgroup, whereas the largest ipsilateral lung V20 reduction was observed in the Catalyst/C-RAD subgroup, followed by OSMS. These analyses are exploratory between-study comparisons intended to describe performance variation across SGRT systems for analyzable outcomes (**Supplementary Appendix 9**).

## Discussion

### Principal findings

In this systematic review and meta-analysis of 52 studies, SGRT/OSMS-assisted DIBH was associated with lower heart Dmean, LAD Dmean, ipsilateral lung Dmean, and ipsilateral lung V20 than FB, with secondary dose-volume outcomes showing broadly consistent directions of effect. The reduction in heart Dmean is clinically relevant because cardiac exposure during breast radiotherapy is associated with subsequent coronary events (3). The ipsilateral lung V20 estimate, however, was model dependent in the sensitivity analyses and should be interpreted cautiously. The comparative image-verified setup analyses showed no statistically significant pooled between-group differences in any direction. Accordingly, the central evidence in this review concerns the dosimetric benefit of the SGRT/OSMS-assisted DIBH workflow, whereas the setup findings primarily describe its implementation performance.

### Relation to previous evidence

Our dosimetric findings are consistent with previous meta-analyses of DIBH versus FB in left-sided breast radiotherapy, including broad systematic reviews and a recent synthesis focused on VMAT (6, 8, 10). Earlier evidence generally shows reduced cardiac, LAD, and ipsilateral lung exposure, although the absolute magnitude of benefit varies with target extent, planning technique, and fractionation. Primary studies using surface-guided DIBH have likewise reported lower cardiopulmonary doses than FB while demonstrating substantial interpatient variation in the magnitude of benefit (23, 28, 30, 31).

### Role of SGRT within the DIBH workflow

This review evaluates SGRT/OSMS-assisted DIBH as implemented in clinical workflows. DIBH changes thoracic geometry by increasing lung volume and moving the heart away from tangential fields, whereas surface guidance supports reproducible external positioning and maintenance of the breath-hold window. The included studies indicate that the magnitude of dose reduction is influenced by chest wall motion, breath-hold reproducibility, target extent, and the spatial relationship of the heart/LAD to the treatment field (23, 26, 28, 31). Current professional guidance positions SGRT as a tool for patient positioning and real-time motion monitoring and emphasizes commissioning, quality assurance, and locally validated image verification workflows (12, 13).

### Technical heterogeneity across SGRT systems and implementations

SGRT/OSMS systems differ substantially in surface-tracking algorithms, reference region configuration, real-time response latency, calibration frequency, and quality control workflows. These technical differences may affect setup verification precision and clinical workflow efficiency and may indirectly influence dosimetric outcomes through breath-hold reproducibility and treatment delivery. Treatment-couch position or rotation can alter the geometry between the patient and the SGRT cameras and affect ROI visibility; gantry rotation, particularly at large angles used for tangential fields, can reduce the effective field of view and visible surface through occlusion by the gantry head or onboard imaging hardware. Both mechanisms may affect surface registration and real-time tracking stability and therefore represent potential sources of technical heterogeneity in setup accuracy. The included studies suggest that these effects are strongly configuration dependent: some ring gantry or closed-bore systems may exhibit local surface occlusion or reduced visible area, whereas optimized camera arrangements can substantially mitigate this problem in other systems. Because the primary studies did not provide standardized couch- or gantry-angle-specific error data, no separate quantitative adjustment or angle-stratified analysis was performed; these factors were instead incorporated into the technical characteristics table and interpretation. Exploratory SGRT-system-stratified analyses showed a dose reduction direction for both heart Dmean and ipsilateral lung V20 across analyzable system subgroups, although effect magnitudes differed. The largest heart Dmean reduction was observed in the OSMS subgroup, whereas the largest ipsilateral lung V20 reduction was observed in the Catalyst/C-RAD subgroup, followed by OSMS. These findings suggest that different SGRT systems and technical implementations may be associated with performance variation in selected dosimetric outcomes. Because the analyses are exploratory between-study comparisons rather than direct head-to-head system comparisons, they are interpreted as evidence of technical heterogeneity. Outcomes that did not provide at least three comparable independent studies per SGRT-system subgroup were not subjected to unstable stratified estimation.

Respiratory-baseline or reference-surface drift may cause the external surface displayed by SGRT to progressively diverge from bony or internal anatomy even when the surface signal remains within the gating/action tolerance. Gradual patient relaxation, changes in respiratory baseline, postural or soft tissue changes, and failure to update the reference surface after image-guided correction can all contribute to this mismatch. Some primary studies mitigated this risk by re-establishing the baseline or reacquiring the reference surface, but such procedures were not consistent across studies. Baseline/reference-surface drift should therefore be considered a potential source of measurement bias when interpreting image-verified residual setup errors and between-study heterogeneity.

### Clinical interpretation of setup accuracy

Comparative meta-analysis found no statistically significant between-group difference in residual setup error; this result cannot be interpreted as evidence of equivalence or non-inferiority. In the single-arm analyses, the pooled residual errors were 0.88, 2.02, and 1.13 mm in the LR/LAT, SI/LNG/CC, and AP/VRT directions, respectively. Using approximately ±3 mm as a clinical reference, all three pooled single-axis means were below 3 mm, indicating that the image-verified setup errors under SGRT/OSMS-DIBH were generally within a clinically acceptable low-millimeter range. This finding supports the ability of SGRT to provide reproducible routine external positioning and real-time DIBH monitoring to help the treatment team promptly identify deviations in surface position or breath-hold status and thereby to support inter- and intrafraction setup consistency and stable DIBH delivery. The principal clinical interpretation focuses on positioning discrepancies observed by image verification after SGRT-guided positioning; study-specific pre- versus post-correction measurement timing is detailed in **Supplementary Appendix 9, Table S9.1**, and measurements explicitly obtained after image-guided correction were not interpreted in the same way as the initial setup errors. The 3D vector represents the spatial magnitude obtained by combining displacements across multiple orthogonal axes and is therefore defined differently from single-axis LR/LAT, SI/LNG/CC, and AP/VRT errors; its numerical value is consequently usually larger than a displacement measured along any one axis. Larger 3D-vector values may be influenced by several factors, including the device/system response latency and patient breath-hold cooperation and reproducibility highlighted by the reviewer as well as study-specific error definitions and measurement workflows. The pooled value of 6.21 mm is therefore better interpreted as an overall three-dimensional displacement magnitude rather than as evidence that single-axis SGRT positioning error exceeds clinical tolerance. Because this pooled estimate was based on only two studies, quantitative separation of the contributing factors remains limited.

### Clinical implications

Of greater translational relevance, SGRT can be used to optimize and individualize IGRT workflows. In selected whole-breast DIBH patients whose initial internal-image verification is accurate and whose early fractions demonstrate stable patient-specific positioning and breath-hold reproducibility, SGRT can assume the primary role in routine external positioning and intrafraction real-time monitoring, supporting a reduction in routine imaging frequency and a shift toward periodic or triggered internal imaging. Among the included studies, Laaksomaa et al. reported that residual systematic error with an SGRT-only whole-breast DIBH workflow could be maintained at approximately 3 mm and proposed that IGRT frequency could be substantially reduced (44). Rudat et al. likewise showed good intrafraction and acceptable interfraction accuracy with SGRT, supporting reduced kV-CBCT frequency, shorter treatment time, and lower additional imaging dose (66). In contrast, daily or more frequent internal image verification should be retained for patients undergoing regional nodal irradiation, complex VMAT/SIB, treatment with small PTV margins, anatomical change or baseline/reference-surface drift during treatment, or inadequate concordance between the external surface and internal anatomy on initial imaging; multicenter data for regional nodal DIBH also support the continued use of more stringent image verification workflows (41, 68). The clinical value of SGRT is therefore best summarized as SGRT-led routine positioning and real-time monitoring combined with patient-specific periodic or triggered internal imaging—providing a basis for risk-adapted IGRT de-escalation rather than universal replacement of internal imaging.

### Strengths and limitations

This review analyzed dosimetric and setup accuracy outcomes separately and focused specifically on SGRT/OSMS-assisted DIBH workflows, but several limitations remain. First, the evidence base consisted primarily of dosimetric comparative studies and observational image verification studies, with variation in treatment targets, prescription/fractionation, planning techniques, surface-monitoring systems, outcome definitions, and image verification protocols. Second, SGRT/OSMS systems differ in surface-tracking algorithms, reference-region configuration, real-time response, calibration, and quality control workflows. Baseline/reference-surface drift may produce mismatch between the external surface and bony/internal anatomy, treatment-couch position or rotation may alter patient–camera geometry and ROI visibility, and gantry rotation, particularly at large treatment angles, may reduce the effective field of view through occlusion by the gantry head or onboard imaging hardware. Because these technical factors were not reported consistently across primary studies, no uniform quantitative adjustment or angle-stratified analysis was possible; they were therefore incorporated into the interpretation as sources of technical heterogeneity and potential measurement bias.

In addition, although operational definitions, unit harmonization, and exclusion of non-equivalent outcomes improved comparability, studies could still differ in the superior/inferior boundaries of the heart, the actual delineated LAD length, and the contouring atlas used, potentially altering absolute Dmean and V20 values. Such differences may be particularly important for the small LAD, which is incompletely visualized on CT, and may amplify between-study heterogeneity. Because this review relied on published aggregate data, uniform re-contouring across all studies was not possible. Several pooled estimates showed substantial heterogeneity, small-study-effect signals were detected for heart Dmean and ipsilateral lung V20, and the V20 estimate was model dependent. Finally, dosimetric and geometric surrogate endpoints cannot directly establish improvements in local control, cardiac events, pulmonary toxicity, treatment efficiency, or patient-reported outcomes. The findings are therefore better suited to workflow selection, quality assurance, and future study design than to claims of clinical superiority of SGRT over other DIBH guidance approaches.

## Conclusions

SGRT/OSMS-assisted DIBH was associated with lower cardiac, LAD, and ipsilateral lung doses in left-sided breast radiotherapy. Comparative setup analyses found no statistically significant pooled differences from the comparator workflows reported in the primary studies; this finding does not establish equivalence or non-inferiority. Pooled residual errors across the three single-axis directions were generally in the low-millimeter range, supporting SGRT/OSMS as a tool for routine external positioning and real-time DIBH monitoring. In selected whole-breast DIBH patients with accurate initial internal-image confirmation and stable early-fraction positioning, SGRT may support a reduction in routine imaging frequency and a shift toward periodic or triggered IGRT; patients receiving regional nodal irradiation and complex treatment techniques or showing inadequate setup stability should retain more frequent internal image verification. Future prospective studies should further evaluate patient-specific IGRT de-escalation strategies with respect to treatment accuracy, efficiency, and clinical outcomes.

## Data Availability

The original contributions presented in the study are included in the article/**Supplementary Material**. Further inquiries can be directed to the corresponding author.
