# Supplementary material for: Surface-guided deep-inspiration breath-hold radiotherapy for left-sided breast cancer: a systematic review and meta-analysis of dosimetric outcomes and image-verified setup accuracy
Source: Front Oncol. 2026 Sep 17;16:1936561. doi: 10.3389/fonc.2026.1936561 (PMC13627035; doi:10.3389/fonc.2026.1936561)
Supplement: Supplementary file 1 [file DataSheet1.pdf]

Supplementary Appendix

Surface-guided deep-inspiration breath-hold radiotherapy for left-sided breast cancer: a systematic review and meta-analysis of dosimetric outcomes and image-verified setup accuracy

Xiaoyan Wu, Huaqiong Wang, Peng Wu, Yin Zhang

Table of contents

Appendix 1: Search strategies 2

Appendix 2: Characteristics of included studies 6

Appendix 3: Data preparation and outcome availability 8

Appendix 4: Methodological quality assessment 9

Appendix 5: Heterogeneity and small-study effects 11

Appendix 6: Secondary dosimetric outcomes 15

Appendix 7: Image-verified setup accuracy: comparative analyses 22

Appendix 8: Image-verified setup accuracy: single-arm analyses 26

Appendix 9:SGRT/OSMS technical heterogeneity, dosimetric definitions, and exploratory SGRT-system-stratified analyses 33

Appendix 1. Search strategies

Supplementary note. All four databases were searched from inception through May 21, 2026, with the final update performed on that date. Before deduplication, 913 records were identified from PubMed, 1,302 from Web of Science, 286 from Embase, and 1,314 from CENTRAL, for a total of 3,815 records. Tables S1.1-S1.4 provide the complete line-by-line search histories for each database.

Table S1.1. PubMed search strategy.

#	Searches
1	("Breast Neoplasms"[MeSH Terms] OR breast cancer*[Title/Abstract] OR breast neoplasm*[Title/Abstract] OR breast tumor*[Title/Abstract] OR breast tumour*[Title/Abstract] OR breast carcinoma*[Title/Abstract] OR mammary cancer*[Title/Abstract] OR mammary neoplasm*[Title/Abstract] OR mammary carcinoma*[Title/Abstract])
2	("left-sided"[Title/Abstract] OR "left sided"[Title/Abstract] OR "left breast"[Title/Abstract] OR "left-sided breast"[Title/Abstract] OR "left sided breast"[Title/Abstract] OR "left-sided breast cancer"[Title/Abstract] OR "left sided breast cancer"[Title/Abstract] OR "left chest wall"[Title/Abstract] OR "left-sided chest wall"[Title/Abstract])
3	("Radiotherapy"[MeSH Terms] OR radiotherap*[Title/Abstract] OR "radiation therapy"[Title/Abstract] OR "radiation treatment"[Title/Abstract] OR irradiation[Title/Abstract] OR irradiat*[Title/Abstract] OR "breast irradiation"[Title/Abstract] OR "adjuvant radiotherapy"[Title/Abstract] OR "external beam radiotherapy"[Title/Abstract] OR EBRT[Title/Abstract] OR 3DCRT[Title/Abstract] OR "3D-CRT"[Title/Abstract] OR IMRT[Title/Abstract] OR VMAT[Title/Abstract] OR tomotherapy[Title/Abstract])
4	("deep inspiration breath hold"[Title/Abstract] OR "deep-inspiration breath-hold"[Title/Abstract] OR "deep inspiratory breath hold"[Title/Abstract] OR "deep-inspiratory breath-hold"[Title/Abstract] OR "moderate deep inspiration breath hold"[Title/Abstract] OR "moderate deep-inspiration breath-hold"[Title/Abstract] OR DIBH[Title/Abstract] OR mDIBH[Title/Abstract] OR vDIBH[Title/Abstract] OR "breath hold"[Title/Abstract] OR "breath-hold"[Title/Abstract] OR breathhold[Title/Abstract] OR "inspiration breath hold"[Title/Abstract] OR "inspiratory breath hold"[Title/Abstract] OR "voluntary breath hold"[Title/Abstract] OR "voluntary breath-hold"[Title/Abstract] OR "respiratory gating"[Title/Abstract] OR "gated radiotherapy"[Title/Abstract] OR "gated radiation therapy"[Title/Abstract])
5	("surface guided radiation therapy"[Title/Abstract] OR "surface-guided radiation therapy"[Title/Abstract] OR "surface guided radiotherapy"[Title/Abstract] OR "surface-guided radiotherapy"[Title/Abstract] OR SGRT[Title/Abstract] OR "surface guidance"[Title/Abstract] OR "surface-guided"[Title/Abstract] OR "surface imaging"[Title/Abstract] OR "surface image guidance"[Title/Abstract] OR "surface monitoring"[Title/Abstract] OR "surface tracking"[Title/Abstract] OR "optical surface monitoring"[Title/Abstract] OR "optical surface management"[Title/Abstract] OR "optical surface imaging"[Title/Abstract] OR "optical surface guidance"[Title/Abstract] OR "optical body surface tracing"[Title/Abstract] OR "body surface tracing"[Title/Abstract] OR OSMS[Title/Abstract] OR "optical surface monitoring system"[Title/Abstract] OR AlignRT[Title/Abstract] OR VisionRT[Title/Abstract] OR "Vision RT"[Title/Abstract] OR Catalyst[Title/Abstract] OR "Catalyst HD"[Title/Abstract] OR "C-RAD"[Title/Abstract] OR CRAD[Title/Abstract] OR Sentinel[Title/Abstract] OR "Varian Identify"[Title/Abstract])
6	(dose[Title/Abstract] OR dosimetric*[Title/Abstract] OR dosimetry[Title/Abstract] OR "dose-volume"[Title/Abstract] OR DVH[Title/Abstract] OR "organ at risk"[Title/Abstract] OR "organs at risk"[Title/Abstract] OR OAR[Title/Abstract] OR OARs[Title/Abstract] OR heart[Title/Abstract] OR cardiac[Title/Abstract] OR "mean heart dose"[Title/Abstract] OR MHD[Title/Abstract] OR LAD[Title/Abstract] OR "left anterior descending"[Title/Abstract] OR coronary[Title/Abstract] OR lung[Title/Abstract] OR pulmonary[Title/Abstract] OR "ipsilateral lung"[Title/Abstract])
7	(setup[Title/Abstract] OR "set-up"[Title/Abstract] OR positioning[Title/Abstract] OR "patient positioning"[Title/Abstract] OR "setup error"[Title/Abstract] OR "set-up error"[Title/Abstract] OR "positioning error"[Title/Abstract] OR accuracy[Title/Abstract] OR reproducib*[Title/Abstract] OR verification[Title/Abstract] OR "image verification"[Title/Abstract] OR "image guidance"[Title/Abstract] OR "image-guided"[Title/Abstract] OR IGRT[Title/Abstract] OR CBCT[Title/Abstract] OR "cone beam"[Title/Abstract] OR "cone-beam"[Title/Abstract] OR "cone beam computed tomography"[Title/Abstract] OR MVCT[Title/Abstract] OR "portal imaging"[Title/Abstract] OR EPID[Title/Abstract] OR intrafraction[Title/Abstract] OR "intra-fraction"[Title/Abstract] OR interfraction[Title/Abstract] OR "inter-fraction"[Title/Abstract] OR "vector error"[Title/Abstract] OR "3D vector"[Title/Abstract] OR "three-dimensional vector"[Title/Abstract] OR displacement[Title/Abstract] OR deviation[Title/Abstract] OR "couch shift"[Title/Abstract] OR "treatment time"[Title/Abstract] OR feasib*[Title/Abstract])
8	#6 OR #7
9	#1 AND #2 AND #3 AND #4 AND #5
10	#9 AND #8
11	(review[Publication Type] OR meta-analysis[Publication Type] OR systematic review[Title/Abstract] OR meta-analysis[Title/Abstract])
12	#10 NOT #11

Table S1.2. Web of Science search strategy.

#	Searches
1	TS=("breast cancer*" OR "breast neoplasm*" OR "breast tumor*" OR "breast tumour*" OR "breast carcinoma*" OR "mammary cancer*" OR "mammary neoplasm*" OR "mammary carcinoma*")
2	TS=("left-sided" OR "left sided" OR "left breast" OR "left-sided breast" OR "left sided breast" OR "left-sided breast cancer" OR "left sided breast cancer" OR "left chest wall" OR "left-sided chest wall")
3	TS=(radiotherap* OR "radiation therapy" OR "radiation treatment" OR irradiation OR irradiat* OR "breast irradiation" OR "adjuvant radiotherapy" OR "external beam radiotherapy" OR EBRT OR 3DCRT OR "3D-CRT" OR IMRT OR VMAT OR tomotherapy)
4	TS=("deep inspiration breath hold" OR "deep-inspiration breath-hold" OR "deep inspiratory breath hold" OR "deep-inspiratory breath-hold" OR "moderate deep inspiration breath hold" OR "moderate deep-inspiration breath-hold" OR DIBH OR mDIBH OR vDIBH OR "breath hold" OR "breath-hold" OR breathhold OR "inspiration breath hold" OR "inspiratory breath hold" OR "voluntary breath hold" OR "voluntary breath-hold" OR "respiratory gating" OR "gated radiotherapy" OR "gated radiation therapy")
5	TS=("surface guided radiation therapy" OR "surface-guided radiation therapy" OR "surface guided radiotherapy" OR "surface-guided radiotherapy" OR SGRT OR "surface guidance" OR "surface-guided" OR "surface imaging" OR "surface image guidance" OR "surface monitoring" OR "surface tracking" OR "optical surface monitoring" OR "optical surface management" OR "optical surface imaging" OR "optical surface guidance" OR "optical body surface tracing" OR "body surface tracing" OR OSMS OR "optical surface monitoring system" OR AlignRT OR VisionRT OR "Vision RT" OR Catalyst OR "Catalyst HD" OR "C-RAD" OR CRAD OR Sentinel OR "Varian Identify")
6	TS=(dose OR dosimetric* OR dosimetry OR "dose-volume" OR DVH OR "organ at risk" OR "organs at risk" OR OAR OR OARs OR heart OR cardiac OR "mean heart dose" OR MHD OR LAD OR "left anterior descending" OR coronary OR lung OR pulmonary OR "ipsilateral lung")
7	TS=(setup OR "set-up" OR positioning OR "patient positioning" OR "setup error" OR "set-up error" OR "positioning error" OR accuracy OR reproducib* OR verification OR "image verification" OR "image guidance" OR "image-guided" OR IGRT OR CBCT OR "cone beam" OR "cone-beam" OR "cone beam computed tomography" OR MVCT OR "portal imaging" OR EPID OR intrafraction OR "intra-fraction" OR interfraction OR "inter-fraction" OR "vector error" OR "3D vector" OR "three-dimensional vector" OR displacement OR deviation OR "couch shift" OR "treatment time" OR feasib*)
8	#6 OR #7
9	#1 AND #2 AND #3 AND #4 AND #5
10	#9 AND #8
11	TS=(review OR "systematic review" OR "meta-analysis" OR "meta analysis") OR DT=(Review)
12	#10 NOT #11

Table S1.3. Cochrane CENTRAL search strategy.

#	Searches
1	MeSH descriptor: [Breast Neoplasms] explode all trees OR ("breast cancer*" OR "breast neoplasm*" OR "breast tumor*" OR "breast tumour*" OR "breast carcinoma*" OR "mammary cancer*" OR "mammary neoplasm*" OR "mammary carcinoma*"):ti,ab,kw
2	("left-sided" OR "left sided" OR "left breast" OR "left-sided breast" OR "left sided breast" OR "left-sided breast cancer" OR "left sided breast cancer" OR "left chest wall" OR "left-sided chest wall"):ti,ab,kw
3	MeSH descriptor: [Radiotherapy] explode all trees OR (radiotherap* OR "radiation therapy" OR "radiation treatment" OR irradiation OR irradiat* OR "breast irradiation" OR "adjuvant radiotherapy" OR "external beam radiotherapy" OR EBRT OR 3DCRT OR "3D-CRT" OR IMRT OR VMAT OR tomotherapy):ti,ab,kw
4	("deep inspiration breath hold" OR "deep-inspiration breath-hold" OR "deep inspiratory breath hold" OR "deep-inspiratory breath-hold" OR "moderate deep inspiration breath hold" OR "moderate deep-inspiration breath-hold" OR DIBH OR mDIBH OR vDIBH OR "breath hold" OR "breath-hold" OR breathhold OR "inspiration breath hold" OR "inspiratory breath hold" OR "voluntary breath hold" OR "voluntary breath-hold" OR "respiratory gating" OR "gated radiotherapy" OR "gated radiation therapy"):ti,ab,kw
5	("surface guided radiation therapy" OR "surface-guided radiation therapy" OR "surface guided radiotherapy" OR "surface-guided radiotherapy" OR SGRT OR "surface guidance" OR "surface-guided" OR "surface imaging" OR "surface image guidance" OR "surface monitoring" OR "surface tracking" OR "optical surface monitoring" OR "optical surface management" OR "optical surface imaging" OR "optical surface guidance" OR "optical body surface tracing" OR "body surface tracing" OR OSMS OR "optical surface monitoring system" OR AlignRT OR VisionRT OR "Vision RT" OR Catalyst OR "Catalyst HD" OR "C-RAD" OR CRAD OR Sentinel OR "Varian Identify"):ti,ab,kw
6	(dose OR dosimetric* OR dosimetry OR "dose-volume" OR DVH OR "organ at risk" OR "organs at risk" OR OAR OR OARs OR heart OR cardiac OR "mean heart dose" OR MHD OR LAD OR "left anterior descending" OR coronary OR lung OR pulmonary OR "ipsilateral lung"):ti,ab,kw
7	(setup OR "set-up" OR positioning OR "patient positioning" OR "setup error" OR "set-up error" OR "positioning error" OR accuracy OR reproducib* OR verification OR "image verification" OR "image guidance" OR "image-guided" OR IGRT OR CBCT OR "cone beam" OR "cone-beam" OR "cone beam computed tomography" OR MVCT OR "portal imaging" OR EPID OR intrafraction OR "intra-fraction" OR interfraction OR "inter-fraction" OR "vector error" OR "3D vector" OR "three-dimensional vector" OR displacement OR deviation OR "couch shift" OR "treatment time" OR feasib*):ti,ab,kw
8	#6 OR #7
9	#1 AND #2 AND #3 AND #4 AND #5
10	#9 AND #8
11	("meta analysis" OR "meta-analysis" OR "systematic review" OR review):ti,ab,kw OR review:pt
12	#10 NOT #11

Table S1.4. Embase search strategy.

#	Searches
1	'breast cancer'/exp OR 'breast tumor'/exp OR 'breast neoplasm*':ti,ab,kw OR 'breast cancer*':ti,ab,kw OR 'breast tumor*':ti,ab,kw OR 'breast tumour*':ti,ab,kw OR 'breast carcinoma*':ti,ab,kw OR 'mammary cancer*':ti,ab,kw OR 'mammary neoplasm*':ti,ab,kw OR 'mammary carcinoma*':ti,ab,kw
2	'left-sided':ti,ab,kw OR 'left sided':ti,ab,kw OR 'left breast':ti,ab,kw OR 'left-sided breast':ti,ab,kw OR 'left sided breast':ti,ab,kw OR 'left-sided breast cancer':ti,ab,kw OR 'left sided breast cancer':ti,ab,kw OR 'left chest wall':ti,ab,kw OR 'left-sided chest wall':ti,ab,kw
3	'radiotherapy'/exp OR radiotherap*:ti,ab,kw OR 'radiation therapy':ti,ab,kw OR 'radiation treatment':ti,ab,kw OR irradiation:ti,ab,kw OR irradiat*:ti,ab,kw OR 'breast irradiation':ti,ab,kw OR 'adjuvant radiotherapy':ti,ab,kw OR 'external beam radiotherapy':ti,ab,kw OR ebrt:ti,ab,kw OR 3dcrt:ti,ab,kw OR '3d-crt':ti,ab,kw OR imrt:ti,ab,kw OR vmat:ti,ab,kw OR tomotherapy:ti,ab,kw
4	'deep inspiration breath hold':ti,ab,kw OR 'deep-inspiration breath-hold':ti,ab,kw OR 'deep inspiratory breath hold':ti,ab,kw OR 'deep-inspiratory breath-hold':ti,ab,kw OR 'moderate deep inspiration breath hold':ti,ab,kw OR 'moderate deep-inspiration breath-hold':ti,ab,kw OR dibh:ti,ab,kw OR mdibh:ti,ab,kw OR vdibh:ti,ab,kw OR 'breath hold':ti,ab,kw OR 'breath-hold':ti,ab,kw OR breathhold:ti,ab,kw OR 'inspiration breath hold':ti,ab,kw OR 'inspiratory breath hold':ti,ab,kw OR 'voluntary breath hold':ti,ab,kw OR 'voluntary breath-hold':ti,ab,kw OR 'respiratory gating':ti,ab,kw OR 'gated radiotherapy':ti,ab,kw OR 'gated radiation therapy':ti,ab,kw
5	'surface guided radiation therapy':ti,ab,kw OR 'surface-guided radiation therapy':ti,ab,kw OR 'surface guided radiotherapy':ti,ab,kw OR 'surface-guided radiotherapy':ti,ab,kw OR sgrt:ti,ab,kw OR 'surface guidance':ti,ab,kw OR 'surface-guided':ti,ab,kw OR 'surface imaging':ti,ab,kw OR 'surface image guidance':ti,ab,kw OR 'surface monitoring':ti,ab,kw OR 'surface tracking':ti,ab,kw OR 'optical surface monitoring':ti,ab,kw OR 'optical surface management':ti,ab,kw OR 'optical surface imaging':ti,ab,kw OR 'optical surface guidance':ti,ab,kw OR 'optical body surface tracing':ti,ab,kw OR 'body surface tracing':ti,ab,kw OR osms:ti,ab,kw OR 'optical surface monitoring system':ti,ab,kw OR alignrt:ti,ab,kw OR visionrt:ti,ab,kw OR 'vision rt':ti,ab,kw OR catalyst:ti,ab,kw OR 'catalyst hd':ti,ab,kw OR 'c-rad':ti,ab,kw OR crad:ti,ab,kw OR sentinel:ti,ab,kw OR 'varian identify':ti,ab,kw
6	dose:ti,ab,kw OR dosimetric*:ti,ab,kw OR dosimetry:ti,ab,kw OR 'dose-volume':ti,ab,kw OR dvh:ti,ab,kw OR 'organ at risk':ti,ab,kw OR 'organs at risk':ti,ab,kw OR oar:ti,ab,kw OR oars:ti,ab,kw OR heart:ti,ab,kw OR cardiac:ti,ab,kw OR 'mean heart dose':ti,ab,kw OR mhd:ti,ab,kw OR lad:ti,ab,kw OR 'left anterior descending':ti,ab,kw OR coronary:ti,ab,kw OR lung:ti,ab,kw OR pulmonary:ti,ab,kw OR 'ipsilateral lung':ti,ab,kw
7	setup:ti,ab,kw OR 'set-up':ti,ab,kw OR positioning:ti,ab,kw OR 'patient positioning':ti,ab,kw OR 'setup error':ti,ab,kw OR 'set-up error':ti,ab,kw OR 'positioning error':ti,ab,kw OR accuracy:ti,ab,kw OR reproducib*:ti,ab,kw OR verification:ti,ab,kw OR 'image verification':ti,ab,kw OR 'image guidance':ti,ab,kw OR 'image-guided':ti,ab,kw OR igrt:ti,ab,kw OR cbct:ti,ab,kw OR 'cone beam':ti,ab,kw OR 'cone-beam':ti,ab,kw OR 'cone beam computed tomography':ti,ab,kw OR mvct:ti,ab,kw OR 'portal imaging':ti,ab,kw OR epid:ti,ab,kw OR intrafraction:ti,ab,kw OR 'intra-fraction':ti,ab,kw OR interfraction:ti,ab,kw OR 'inter-fraction':ti,ab,kw OR 'vector error':ti,ab,kw OR '3d vector':ti,ab,kw OR 'three-dimensional vector':ti,ab,kw OR displacement:ti,ab,kw OR deviation:ti,ab,kw OR 'couch shift':ti,ab,kw OR 'treatment time':ti,ab,kw OR feasib*:ti,ab,kw
8	#6 OR #7
9	#1 AND #2 AND #3 AND #4 AND #5
10	#9 AND #8
11	review:it OR 'meta analysis':ti,ab,kw OR 'meta-analysis':ti,ab,kw OR 'systematic review':ti,ab,kw
12	#10 NOT #11

Appendix 2. Characteristics of included studies

Table S2.1. Characteristics of the 52 included studies.

First author year	Country/Region	Sample size	Clinical stage	Age	Prescription dose/fractions	DIBH guidance/monitoring
Zhang 2023	China	20 (20/20)	0–II	45 (27–63)	43.5 Gy/15F (+SIB 49.5 Gy)	OSMS/AlignRT-DIBH
Zhang 2020	China	48 (48/48)	Early-stage	54 (40–68)	50 Gy/25F (+SIB 60 Gy)	OSMS/Catalyst-DIBH
Abdollahi 2022	Iran	20 (20/20)	NR	NR	50 Gy/25F (+10 Gy boost)	Catalyst/Sentinel-DIBH
Schönecker 2016	Germany	13 (13/13)	NR	46.9 (36–63)	50 Gy/25F	Catalyst/Sentinel-DIBH
Huang Chengjian 2020	China	16 (8/8)	NR	42 (35–50)	NR	OSMS-DIBH
Wolf 2023	Germany	130 (130/130)	T1–T2	NR	40.05 Gy/15F or 50–50.4 Gy/25–28F	Sentinel/Catalyst-DIBH
Kügele 2018	Sweden	40 (40/40)	NR	46/59	50 Gy/25F	Catalyst-DIBH
Mkanna 2018	Lebanon	103 (103/103)	T0–T4	51.9 ± 11.8	50 Gy/25F	AlignRT-DIBH
Tanguturi 2015	United States	146 (146/146)	0–IV	49.5/58	50 Gy/25F	AlignRT-DIBH
Wu 2023a	China	98 (98/98)	NR	44 (26–64)	40.5 Gy/15F	Sentinel-DIBH
Schönecker 2024	Germany/Italy	585 (585/585)	Tis–T3, N0–N3	57 (24–84)	40.05 Gy/15F or 50 Gy/25F	Catalyst-DIBH
Rudat 2024a	China	26 (26/26)	T0–T4, N0–N3	46 (29–66)	40.05 Gy/15F or 40 Gy/16F (+boost)	SGRT-DIBH
Kadhim 2024	Sweden	20 (20/20)	NR	59 (45–77)	50 Gy/25F	VitalHold/Catalyst HD SGRT-DIBH
Zeng 2023	United States	60 (20/40)	Early/locally advanced	58 (34–86)	26–50 Gy/5–25F	SGRT/RPM-DIBH
Li 2021	China	15 (15/15)	NR	≤40/40–60/≥60	43.5 Gy/15F	ABC+Sentinel-DIBH
Eber 2023	Switzerland	10 (10/10)	pT1–2N0	63 (48–80)	40.05 Gy/15F	AlignRT-DIBH
Vuong 2018	Canada	29 (29/29)	IA–IIB	54 (32–80)	42.56–50.4 Gy/16–28F (+boost)	AlignRT-DIBH
Rochet 2015	Germany	35 (35/35)	pTis–pT3, N0–N1	51 (35–69)	42.4–50.4 Gy/16–28F (+boost)	AlignRT-DIBH
Lu 2023a	United States	40 (20/20)	Early-stage/N+	NR	42.4–50 Gy/16–25F	SGRT/RPM-DIBH
Rossi 2022	Finland	51 single-arm	NR	NR	50 Gy/25F	SGRT-DIBH
Penninkhof 2022	Netherlands	326 (228/98)	NR	NR	40.05–50 Gy/15–25F	AlignRT-DIBH

First author year	Country/Region	Sample size	Clinical stage	Age	Prescription dose/fractions	DIBH guidance/monitoring
Laaksomaa 2022	Finland	141 (45/50/46)	NR	60/57/59	40.05 Gy/15F	AlignRT/Catalyst/RPM-DIBH
Lu 2023b	China	10 single-arm	NR	57 (32–74)	NR	AlignRT OSI + 2DkV/CBCT-DIBH
Hamming 2019	Netherlands	18 single-arm	NR	NR	NR	AlignRT+ABC-DIBH
Laaksomaa 2019	Finland	50 (25/25)	N0	NR	42.56 Gy/16F or 50 Gy/25F	AlignRT/Catalyst-DIBH
Fassi 2018	Italy	10 single-arm	NR	NR	45 Gy/20F (+boost)	Optical tracking-DIBH
Tang 2014	Japan	50 single-arm	NR	NR	32–50 Gy/16–25F	AlignRT-DIBH
Abubakar 2023	Austria	13 (6/7)	I–IV	51 (34–68)	5F or 15–24F (Gy NA)	ToF optical surface camera + CBCT/EPID-DIBH
Wu 2023b	China	60 (30/30)	NR	45.77/45.76	NR	OSMS/Catalyst-DIBH
Dekker 2023	United States	84 single-arm	NR	55 (35–77)	40.05 Gy/15F or 53.4 Gy/20F	Varian Identify SGRT-DIBH
Benkhaled 2022	Belgium	15 single-arm	NR	45.4 (32–63)	40.05 Gy/15F	vDIBH + SGRT tracking + daily CBCT
Rong 2014	United States	18 single-arm	NR	NR	50 Gy/25F	AlignRT+RPM-DIBH
Alaimo 2026	Italy	154 (70/84)	NR	54 ± 9	40.05 Gy/15F or 50 Gy/25F	AlignRT Advance-DIBH
Nankali 2022	Denmark	10 single-arm	N0	50.5 (40–67)	40–42.3 Gy/15–18F (+SIB)	AlignRT/RPM-DIBH
Byrne 2024	Australia	26 analyzable	NR	59 (34–82)	40.05 Gy/15F or 50 Gy/25F	Optical surface tracking/RPM-DIBH
Zhao 2023	China	25 single-arm	NR	NR	NR	Varian Identify SGRT-DIBH
Lorchel 2022	France	22 single-arm	NR	63	50 Gy/25F (+47.5 Gy nodes)	AlignRT InBore-DIBH
Parsons 2023	United States	8 patients	NR	NR	50–50.4 Gy/25–28F	VisionRT SGRT/ABC-DIBH
Delombaerde 2021	Belgium	7 single-arm	NR	68–74	45.57/55.86 Gy/21F	SDX + intra-bore surface tracking-DIBH
Alderliesten 2013	Netherlands	20 single-arm	NR	51 (35–65)	50–64.4 Gy/25–31F	AlignRT-DIBH
Betgen 2013	Netherlands	19 single-arm	NR	50 (35–65)	50–66 Gy/25–33F	AlignRT-DIBH
Guo 2026	China	20 (10/10)	T1–T3N0M0	43.5/46.5	42.56 Gy/16F (+8.7 Gy/3F boost)	SGRT/RPM-DIBH
Han 2026	Korea	20 (9/11)	NR	NR	43.2 Gy/16F	ExacTrac Dynamic SGRT-DIBH
Järvinen 2025	Finland	50 (25/25)	NR	NR	NR	Sentinel/Catalyst-DIBH

First author year	Country/Region	Sample size	Clinical stage	Age	Prescription dose/fractions	DIBH guidance/monitoring
Chan 2025	Canada	55 (20/19/16)	NR	55	40.05–50 Gy/15–25F	SGRT/ABC/vDIBH
Lai 2024	China	124 (39/36/49)	NR	52.2 (22–72)	NR	Sentinel/Catalyst SGRT-DIBH
Rudat 2024b	China	23 single-arm	pT/ypT0–3, N0–N2	NR	40.05 Gy/15F; 40/48 Gy/16F; 50.4 Gy/28F	AlignRT-DIBH
Buschmann 2024	Austria	30 single-arm	NR	56 (40–78)	40.05 Gy/15F (+10 Gy/4F boost)	ExacTrac Dynamic thermo-optical SGRT-DIBH
Laaksomaa 2024	Finland/Sweden	150 (50/100)	NR	59–60	40.05 Gy/15F or 50 Gy/25F	AlignRT/Catalyst-DIBH
Bao 2025	China	30 (30/30)	NR	43.5 ± 9.14	NR	Sentinel/Catalyst+CBCT-DIBH
Crop 2023	France	855/2401 fractions	NR	NR	40.05–60 Gy/15–25F; 52.2 Gy/18F	Catalyst/Sentinel-DIBH
Liu 2023	China	70 (35/35)	NR	43.2/45.9	NR	C-RAD optical body surface tracing/ABC-DIBH

Note. Clinical and technical characteristics are reported as available in the source publications. Numbers in parentheses are interpreted according to the original study design. For paired planning data generated from the same patients under two conditions (e.g., 20 [20/20]), 20 patients contributed 20 DIBH and 20 FB conditions/plans; this does not represent 40 independent patients. For multigroup studies (e.g., 141 [45/50/46]), the values in parentheses indicate the sample sizes of the individual study groups. When the analytical unit was a plan, image, or treatment fraction rather than a patient, this is stated explicitly in the table. DIBH = deep-inspiration breath-hold; SIB = simultaneous integrated boost; NR = not reported; SGRT = surface-guided radiotherapy; OSMS = optical surface monitoring system; FB = free breathing.

Appendix 3. Data preparation and outcome availability

Table S3.1. Outcome availability, units, and source of dispersion data.

Analysis	Outcome	Unit	Studies	Effects	Total n	Directly reported SD rows	IQR-derived SD rows
Dose double-arm	Heart Dmax	Gy	11	16	1876	0	16
Dose double-arm	Heart Dmean	Gy	18	23	2552	0	23
Dose double-arm	Heart V10	%	5	5	456	3	2
Dose double-arm	Heart V20	%	4	4	360	2	2
Dose double-arm	Heart V30	%	3	3	568	1	2
Dose double-arm	Heart V5	%	6	6	788	3	3
Dose double-arm	Ipsilateral/left lung Dmean	Gy	12	15	1944	0	15
Dose double-arm	Ipsilateral/left lung V20	%	12	13	1062	4	9
Dose double-arm	Ipsilateral/left lung V5	%	5	5	770	2	3
Dose double-arm	LAD Dmax	Gy	8	14	3204	0	14
Dose double-arm	LAD Dmean	Gy	10	13	1854	0	13
Setup single-arm	3D vector error	mm	5	6	1249	1	5
Setup single-arm	AP/VRT error	mm	16	54	2757	3	51
Setup single-arm	LR/LAT error	mm	13	41	2134	2	39
Setup single-arm	SI/LNG/CC error	mm	14	81	3651	2	79
Setup two-arm	3D vector error	mm	2	2	40	0	2
Setup two-arm	AP/VRT error	mm	19	53	10716	0	53
Setup two-arm	LR/LAT error	mm	17	49	10306	0	49
Setup two-arm	SI/LNG/CC error	mm	17	88	13297	0	88

Note. When a primary study reported dispersion as an interquartile range, range, standard error, confidence interval, or another convertible form, it was converted using the prespecified method. The table records the source and handling of dispersion data ultimately used for each outcome. LAD = left anterior descending coronary artery; LR/LAT = left-right/lateral; SI/LNG/CC = superior-inferior/longitudinal/craniocaudal; AP/VRT = anterior-posterior/vertical.

Appendix 4. Methodological quality assessment

Table S4.1. Study-level methodological quality assessment using the adapted NOS-based tool.

Study	Total score	Rating
Zhang 2023	9	Higher
Zhang 2020	8	Higher
Abdollahi 2022	7	Higher
Schönecker 2016	7	Higher
Huang Chengjian 2020	7	Higher
Wolf 2023	8	Higher
Kügele 2018	7	Higher
Mkanna 2018	9	Higher
Tanguturi 2015	9	Higher
Wu 2023a	7	Higher
Schönecker 2024	9	Higher
Rudat 2024a	7	Higher
Kadhim 2024	7	Higher
Zeng 2023	8	Higher
Li 2021	7	Higher
Eber 2023	7	Higher
Vuong 2018	9	Higher
Rochet 2015	9	Higher
Lu 2023a	8	Higher
Rossi 2022	8	Higher
Penninkhof 2022	7	Higher
Laaksomaa 2022	7	Higher
Lu 2023b	8	Higher
Hamming 2019	8	Higher
Laaksomaa 2019	7	Higher
Fassi 2018	8	Higher
Tang 2014	8	Higher

Study	Total score	Rating
Abubakar 2023	8	Higher
Wu 2023b	7	Higher
Dekker 2023	8	Higher
Benkhaled 2022	8	Higher
Rong 2014	8	Higher
Alaimo 2026	7	Higher
Nankali 2022	8	Higher
Byrne 2024	8	Higher
Zhao 2023	8	Higher
Lorchel 2022	8	Higher
Parsons 2023	6	Moderate
Delombaerde 2021	6	Moderate
Alderliesten 2013	7	Higher
Betgen 2013	7	Higher
Guo 2026	6	Moderate
Han 2026	6	Moderate
Järvinen 2025	7	Higher
Chan 2025	7	Higher
Lai 2024	7	Higher
Rudat 2024b	6	Moderate
Buschmann 2024	6	Moderate
Laaksomaa 2024	7	Higher
Bao 2025	6	Moderate
Crop 2023	6	Moderate
Liu 2023	7	Higher

Table S4.2. Nine-item scoring criteria for the adapted NOS-based tool.

Domain	Item	Criterion for 1 point	Criterion for 0 points / unclear
Selection	1. Representativeness of the study population	Study population was relevant to the target left-breast/chest-wall radiotherapy population, with a clear source and eligibility criteria	Population source was unclear or obviously selective and could not be judged
Selection	2. Definition of eligibility/clinical context	Left-sided breast cancer, treatment extent, and key clinical	Key information was missing

Domain	Item	Criterion for 1 point	Criterion for 0 points / unclear
		context were adequately described	
Selection	3. Ascertainment of the SGRT/OSMS-DIBH workflow	SGRT/OSMS was clearly involved in positioning, DIBH, or surface monitoring/verification	Role of SGRT/OSMS was unclear
Selection	4. Adequacy of comparator/paired condition	Dosimetric comparison had an interpretable FB/planning comparator, or the single-arm setup cohort was clearly defined	Comparator/reference condition was unclear
Comparability	5. Comparability or control of key planning/treatment factors	Key factors such as planning technique, target volume, and prescription were identical, paired, or reasonably controlled	Key differences were uncontrolled or not described
Comparability	6. Comparability or adequate description of setup/workflow factors	Key factors such as immobilization, ROI, image correction, and breath-hold workflow were adequately described	Key workflow factors were insufficiently described for judgment
Outcome/reporting	7. Objective outcome assessment	Dose was derived from DVH/TPS; setup was assessed objectively using CBCT, kV, MV/EPID, stereoscopic X-ray, or a validated surface system	Method used to assess the primary outcome was unclear
Outcome/reporting	8. Completeness of quantitative reporting	Sample size, measure of central tendency, and available dispersion/error definition were sufficient for interpretation or pooling	Key numerical data were missing and could not be interpreted
Outcome/reporting	9. Completeness of verification/outcome reporting	Dose or image-verification workflow and main outcomes were reported with reasonable completeness, without obvious unexplained selective missingness	Major verification/outcome reporting was clearly insufficient or unclear

Note. Because the included evidence consisted primarily of non-randomized dosimetric comparison studies and image-verification studies, some conventional NOS items did not fully capture the planning-comparison and image-verification features of this evidence base. The corresponding decision criteria were therefore contextually adapted within the existing NOS framework. The adapted tool retains nine items and is used to describe methodological quality in a structured manner; scores are not used to weight pooled effect estimates.

Note. Total scores of 7-9 were classified as higher methodological quality and scores of 4-6 as moderate methodological quality. The complete nine-item decision rules are provided in Table S4.2. Scores were used primarily to describe methodological quality and for sensitivity analyses restricted to higher-quality studies.

Appendix 5. Heterogeneity and small-study effects

Table S5.1. Heterogeneity and 95% prediction intervals for estimable outcomes.

Outcome	Analysis type	k	I2 (%)	95% prediction interval
Heart Dmean	Dose double-arm	18	84.5	-2.27 to -0.15
LAD Dmean	Dose double-arm	10	93.8	-15.10 to 1.00
Ipsilateral/left lung Dmean	Dose double-arm	12	72.2	-2.39 to 0.62
Ipsilateral/left lung V20	Dose double-arm	12	93.6	-5.57 to 0.98
Heart Dmax	Dose double-arm	11	97.0	-36.25 to 6.54
Heart V5	Dose double-arm	6	91.2	-9.92 to -1.55
Heart V10	Dose double-arm	5	61.4	-6.51 to -0.99
Heart V20	Dose double-arm	4	12.6	-2.07 to -1.24
Heart V30	Dose double-arm	3	71.4	-4.07 to 1.38
LAD Dmax	Dose double-arm	8	96.7	-44.88 to 4.65
Ipsilateral/left lung V5	Dose double-arm	5	83.6	-10.66 to 5.32
3D vector error	Setup double-arm	2	88.5	NE
LR/LAT error	Setup double-arm	14	79.8	-1.13 to 0.94
SI/LNG/CC error	Setup double-arm	14	94.1	-2.23 to 1.94
AP/VRT error	Setup double-arm	15	93.6	-1.79 to 1.33
3D vector error	Setup single-arm	2	97.0	NE
LR/LAT error	Setup single-arm	7	80.9	-0.42 to 2.19
SI/LNG/CC error	Setup single-arm	7	84.9	0.05 to 3.99
AP/VRT error	Setup single-arm	7	68.8	0.25 to 2.01

Note. Dosimetric effects are mean differences (MDs) for SGRT/OSMS-assisted DIBH minus FB. Setup outcomes are expressed as either comparative MDs or single-arm pooled means, according to the analysis type. NE = not estimable.

Table S5.2. Small-study effect assessment for primary dosimetric outcomes.

Outcome	Test	Statistic	P value
Heart Dmean	Egger regression	-3.67	0.002
LAD Dmean	Egger regression	-1.88	0.097
Ipsilateral/left lung Dmean	Egger regression	-0.57	0.580
Ipsilateral/left lung V20	Egger regression	-4.19	0.002

Note. Formal tests of funnel-plot asymmetry should be interpreted cautiously when few studies contribute to an outcome.

Table S5.3. Sensitivity analyses for primary dosimetric outcomes.

Outcome	Main random-effects estimate	Common-effect estimate	Additional sensitivity analysis
Heart Dmean	MD -1.21 (-1.48 to -0.93)	MD -0.80 (-0.86 to -0.73)	Excluding small studies: MD -1.14 (-1.47 to -0.82)
LAD Dmean	MD -7.05 (-9.39 to -4.71)	MD -4.44 (-4.85 to -4.02)	Excluding small studies: MD -7.21 (-10.92 to -3.51)
Ipsilateral/left lung Dmean	MD -0.88 (-1.34 to -0.42)	MD -0.77 (-0.92 to -0.62)	Excluding small studies: MD -1.01 (-1.64 to -0.39)
Ipsilateral/left lung V20	MD -2.30 (-3.27 to -1.32)	MD -0.02 (-0.05 to 0.02)	Directly reported SD only: MD -2.79 (-3.29 to -2.28)

Note. All estimates are MDs (95% CIs) for SGRT/OSMS-assisted DIBH minus FB. The common-effect estimate for ipsilateral lung V20 was close to the null, whereas point estimates in the remaining sensitivity analyses remained negative.

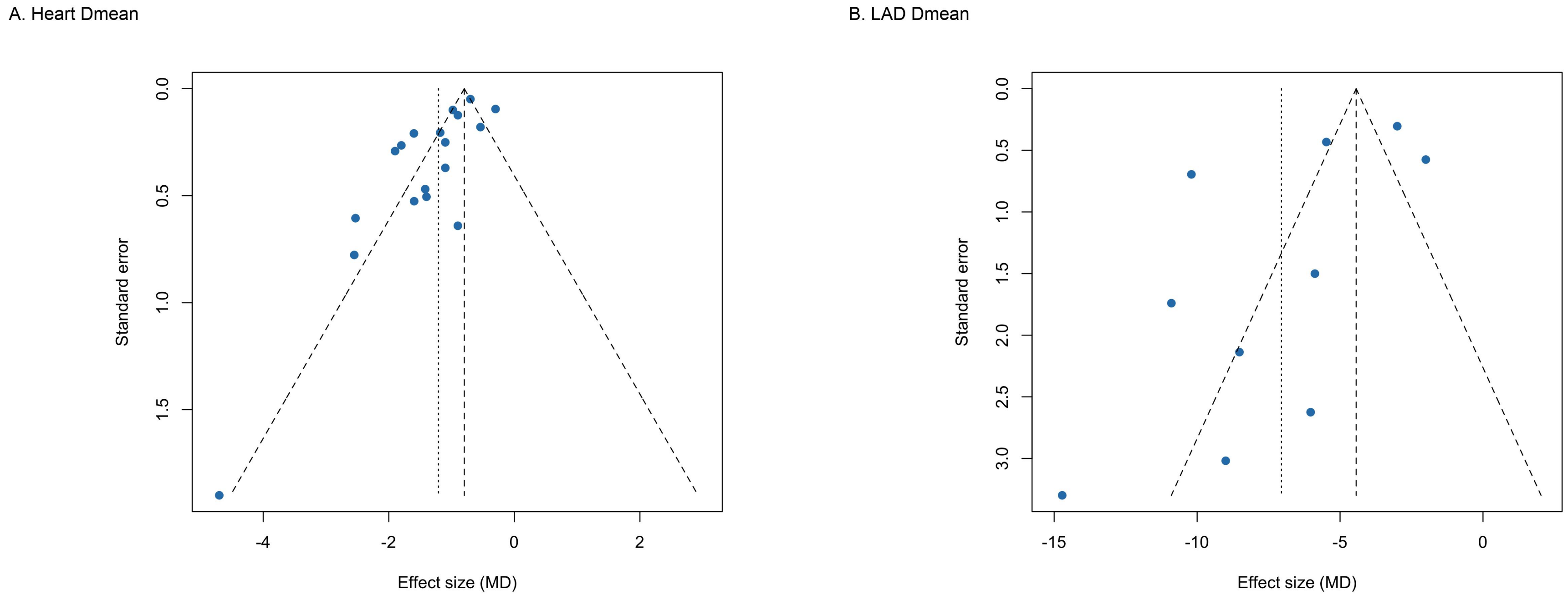

Figure S5.1. Funnel plots for Heart Dmean and LAD Dmean. LAD = left anterior descending coronary artery; Dmean = mean dose.

A. Ipsilateral/left lung Dmean

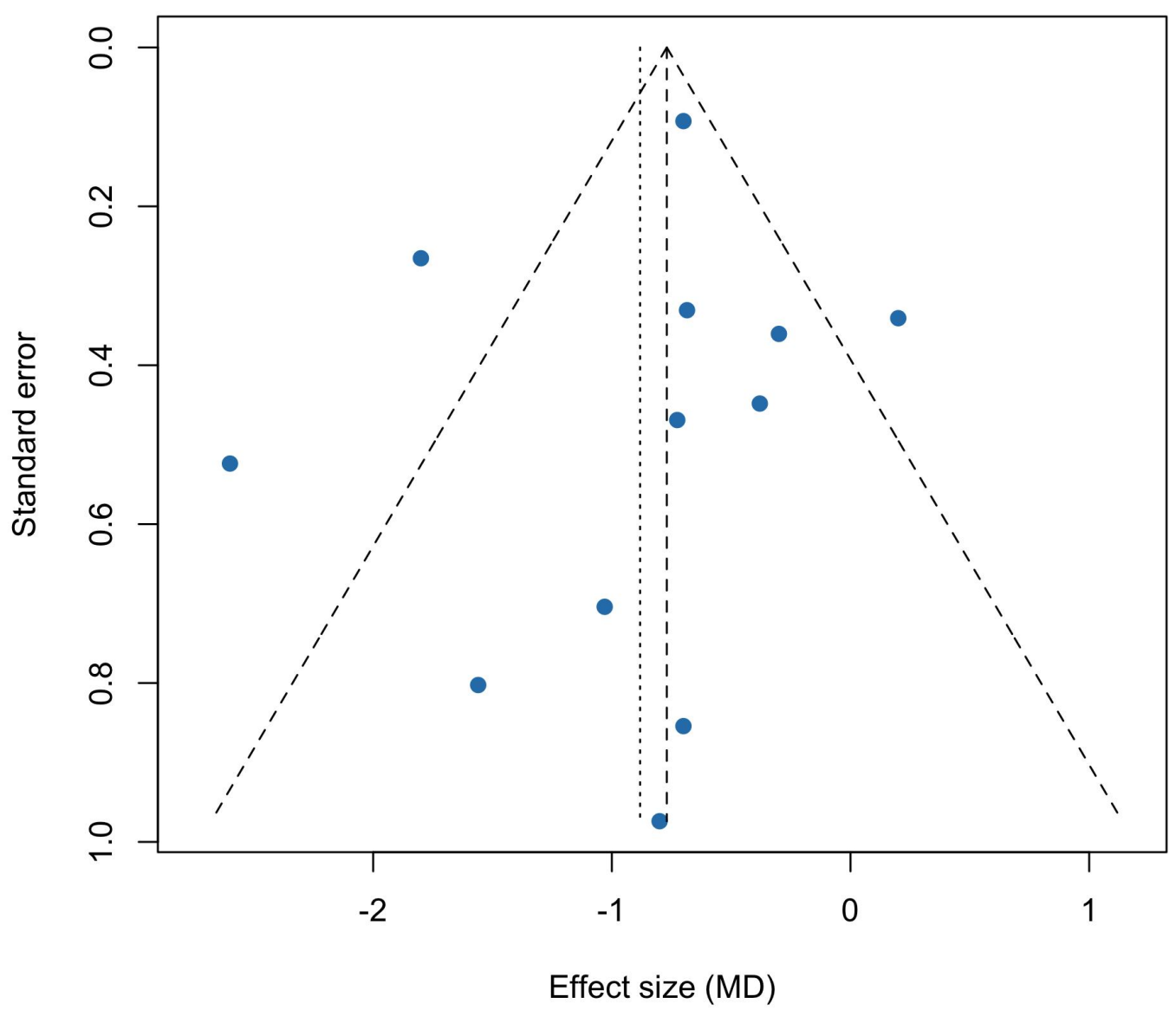

B. Ipsilateral/left lung V20

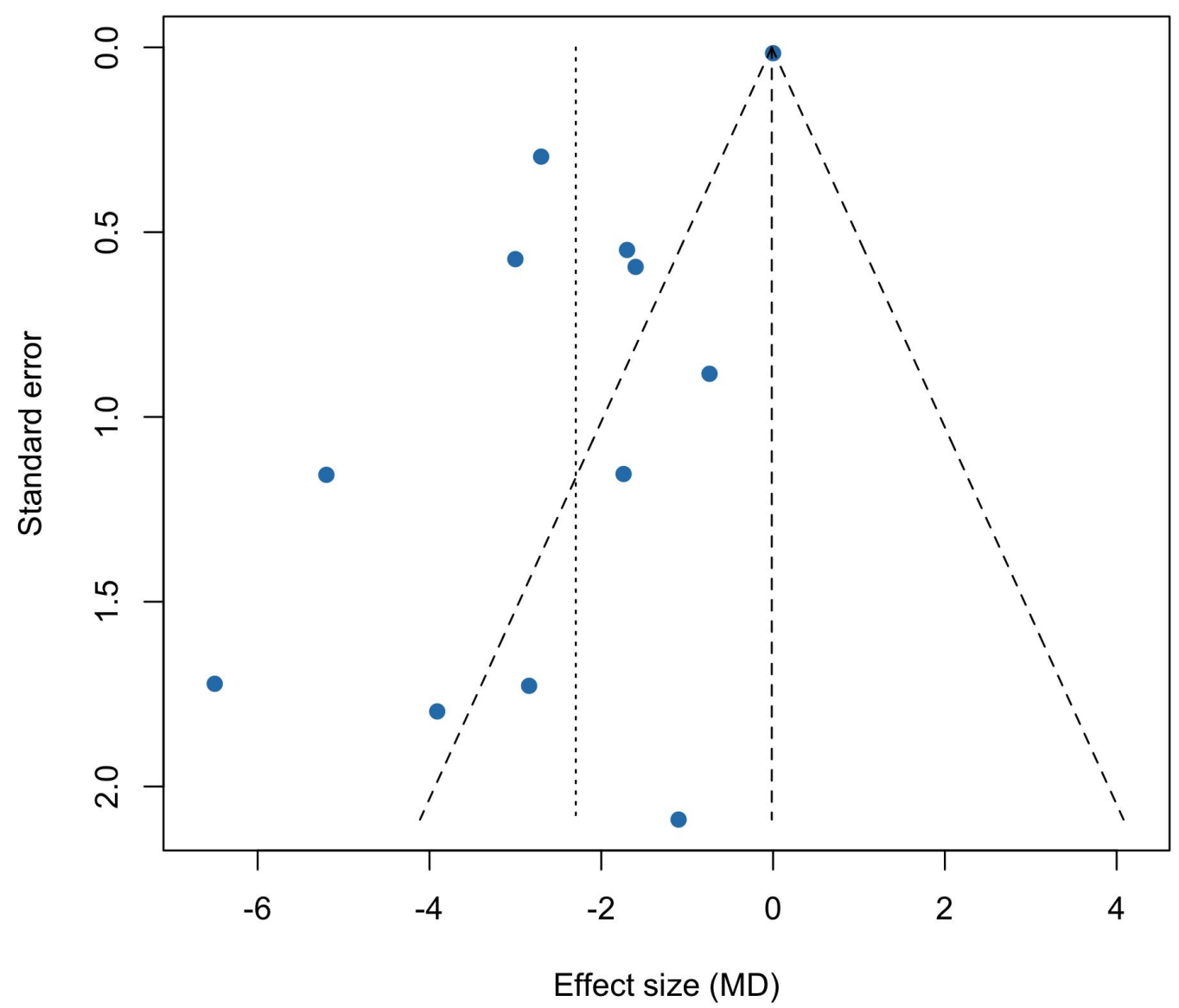

Figure S5.2. Funnel plots for ipsilateral lung Dmean and V20.

Appendix 6. Secondary dosimetric outcomes

Forest plots for secondary dosimetric outcomes are shown below. Dosimetric MDs are defined as SGRT/OSMS-assisted DIBH minus FB; negative values indicate lower dose with SGRT/OSMS-assisted DIBH.

	Experimental			Control					
**Study**	**n.e**	**mean.e**	**SD.e**	**n.c**	**mean.c**	**SD.c**	**MD**	**95% CI**	**Weight**
Eber 2023	10	20.30	15.6000	10	37.10	7.9000	-16.80	[-27.64; -5.96]	7.9%
Kadhim 2024	20	3.20	1.3250	20	6.40	4.3000	-3.20	[-5.17; -1.23]	10.7%
Kügele 2018	20	2.70	2.6000	20	6.98	11.3475	-4.28	[-9.38; 0.82]	10.0%
Rochet 2015	35	24.40	14.2000	35	52.50	5.9000	-28.10	[-33.19; -23.01]	10.0%
Rong 2014	1	15.10	11.2688	1	38.20	11.2688	-23.10	[-54.33; 8.13]	2.7%
Schönecker 2016	9	19.74	15.5200	9	47.90	1.3900	-28.16	[-38.34; -17.98]	8.2%
Schönecker 2024	367	19.85	11.7600	367	34.22	8.6300	-14.37	[-15.86; -12.88]	10.8%
Vuong 2018	29	28.33	10.6295	29	43.70	10.6295	-15.37	[-20.84; -9.90]	9.9%
Wolf 2023	130	21.20	8.7333	130	46.10	7.7500	-24.90	[-26.91; -22.89]	10.7%
Zeng 2023	10	31.88	9.5200	10	39.54	12.7600	-7.66	[-17.53; 2.21]	8.3%
Zhang 2020	48	13.40	4.7000	48	19.30	6.1000	-5.90	[-8.08; -3.72]	10.7%
**Common effect model**	**679**			**679**			**-13.01**	**[-13.89; -12.13]**	**.**
**Random effects model**							**-14.86**	**[-20.75; -8.97]**	**100.0%**
**Prediction interval**								**[-36.25; 6.54]**	

Heterogeneity: $I^2 = 97.0\%$, $\tau^2 = 83.1665$, $p < 0.0001$

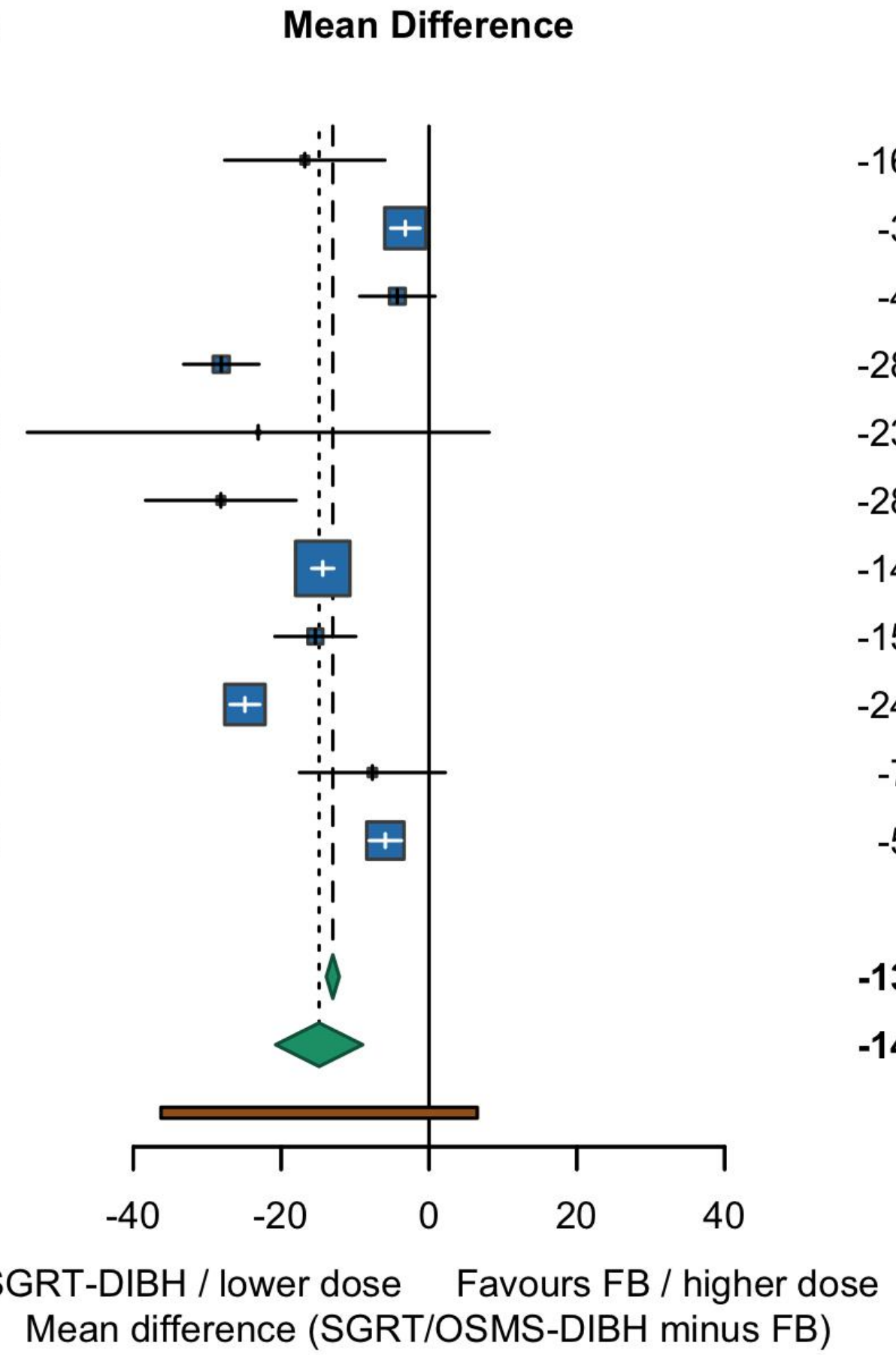

Figure S6.1. Forest plot for Heart Dmax.

Study	Experimental n.e	Experimental mean.e	Experimental SD.e	Control n.c	Control mean.c	Control SD.c	MD	95% CI	Weight
Tanguturi 2015	146	3.80	6.8667	146	8.00	7.3667	-4.20	[-5.83; -2.57]	19.9%
Rochet 2015	35	1.50	0.2000	35	8.20	0.7000	-6.70	[-6.94; -6.46]	26.3%
Rudat 2024	26	2.90	4.8000	26	12.80	13.7000	-9.90	[-15.48; -4.32]	5.4%
Schönecker 2016	9	1.18	0.7700	9	6.75	4.3900	-5.57	[-8.48; -2.66]	12.9%
Wolf 2023	130	0.70	1.6667	130	4.80	3.7500	-4.10	[-4.81; -3.39]	25.0%
Zhang 2020	48	15.90	9.0000	48	24.10	8.6000	-8.20	[-11.72; -4.68]	10.4%
**Common effect model**	**394**			**394**			**-6.39**	**[-6.62; -6.17]**	**.**
**Random effects model**							**-5.74**	**[-7.20; -4.28]**	**100.0%**
**Prediction interval**								**[-9.92; -1.55]**	

Heterogeneity: $I^2 = 91.2\%$, $\tau^2 = 2.0928$, $p < 0.0001$

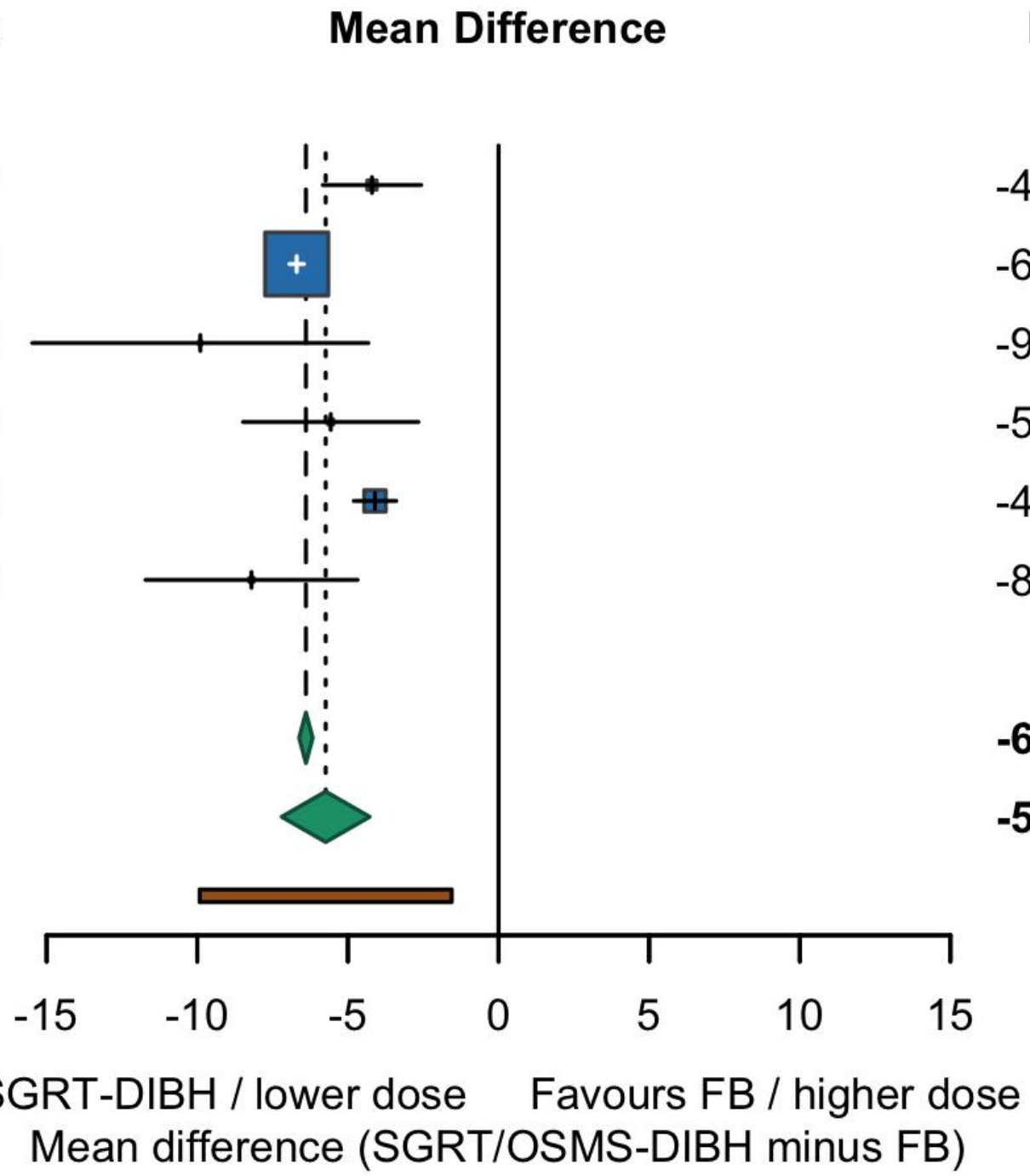

Figure S6.2. Forest plot for Heart V5.

	Experimental			Control					
**Study**	**n.e**	**mean.e**	**SD.e**	**n.c**	**mean.c**	**SD.c**	**MD**	**95% CI**	**Weight**
Li 2021	15	5.00	3.8000	15	9.40	5.2000	-4.40	[-7.66; -1.14]	8.1%
Rudat 2024	26	0.70	1.1250	26	5.40	4.0750	-4.70	[-6.32; -3.08]	20.2%
Schönecker 2016	9	0.26	0.3900	9	4.12	3.4500	-3.86	[-6.13; -1.59]	13.8%
Wolf 2023	130	0.10	1.0500	130	2.80	2.1667	-2.70	[-3.11; -2.29]	37.5%
Zhang 2020	48	4.30	2.7000	48	8.70	5.0000	-4.40	[-6.01; -2.79]	20.4%
**Common effect model**	**228**			**228**			**-2.96**	**[-3.34; -2.58]**	**.**
**Random effects model**							**-3.75**	**[-4.79; -2.71]**	**100.0%**
**Prediction interval**								**[-6.51; -0.99]**	

Heterogeneity: $I^2 = 61.4\%$, $\tau^2 = 0.7090$, $p = 0.0348$

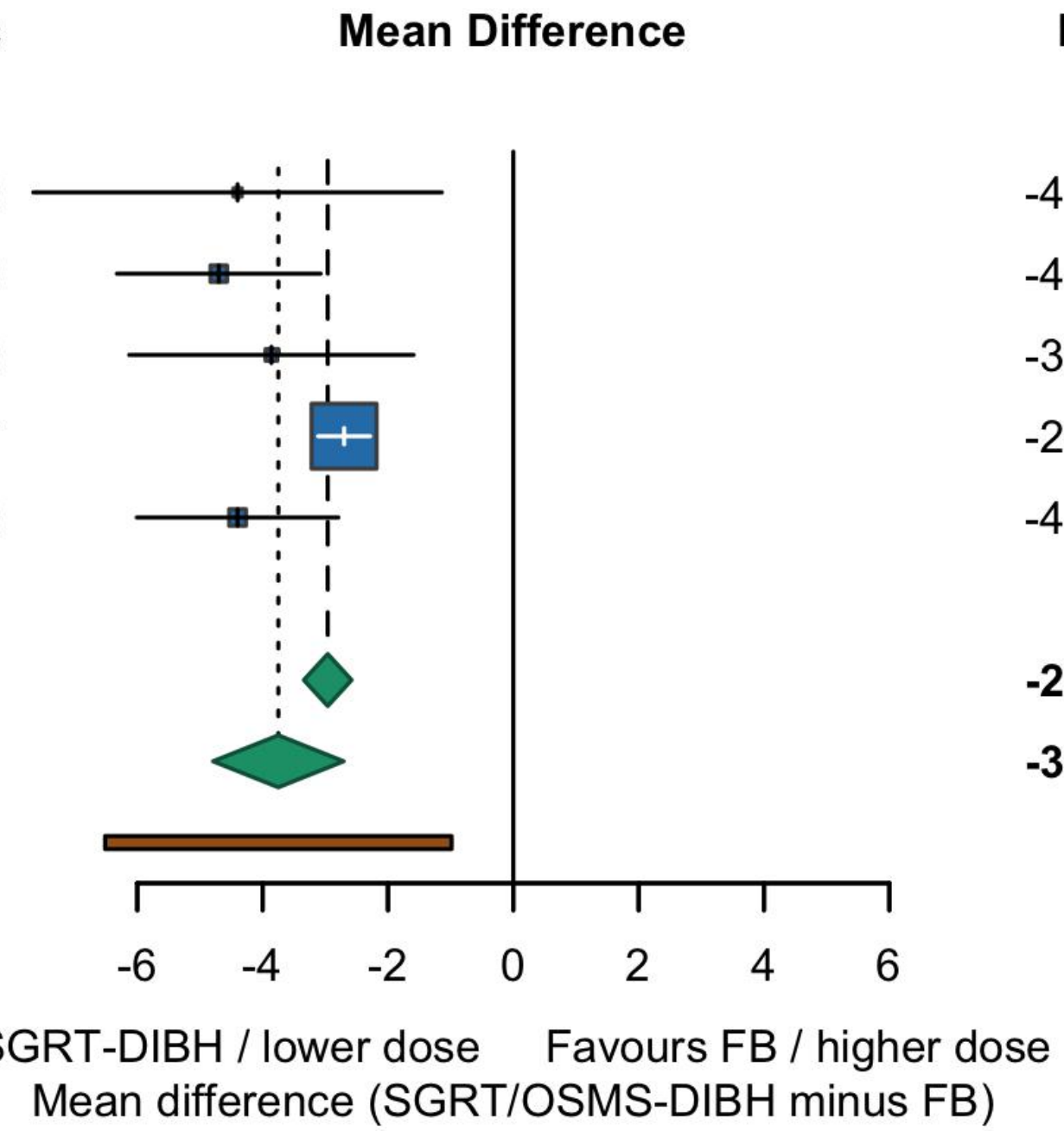

Figure S6.3. Forest plot for Heart V10.

Study	Experimental n.e	Experimental mean.e	Experimental SD.e	Control n.c	Control mean.c	Control SD.c	MD	95% CI	Weight
Li 2021	15	2.30	2.0000	15	5.40	3.6000	-3.10	[-5.18; -1.02]	1.5%
Rudat 2024	26	0.00	0.5000	26	1.70	2.0000	-1.70	[-2.49; -0.91]	10.5%
Schönecker 2016	9	0.09	0.1800	9	2.92	2.9500	-2.83	[-4.76; -0.90]	1.8%
Wolf 2023	130	0.00	0.7000	130	1.60	1.4500	-1.60	[-1.88; -1.32]	86.2%
**Common effect model**	**180**			**180**			**-1.66**	**[-1.91; -1.40]**	**.**
**Random effects model**							**-1.66**	**[-1.91; -1.40]**	**100.0%**
**Prediction interval**								**[-2.07; -1.24]**	

Heterogeneity: $I^2 = 12.6\%$, $\tau^2 < 0.0001$, $p = 0.3295$

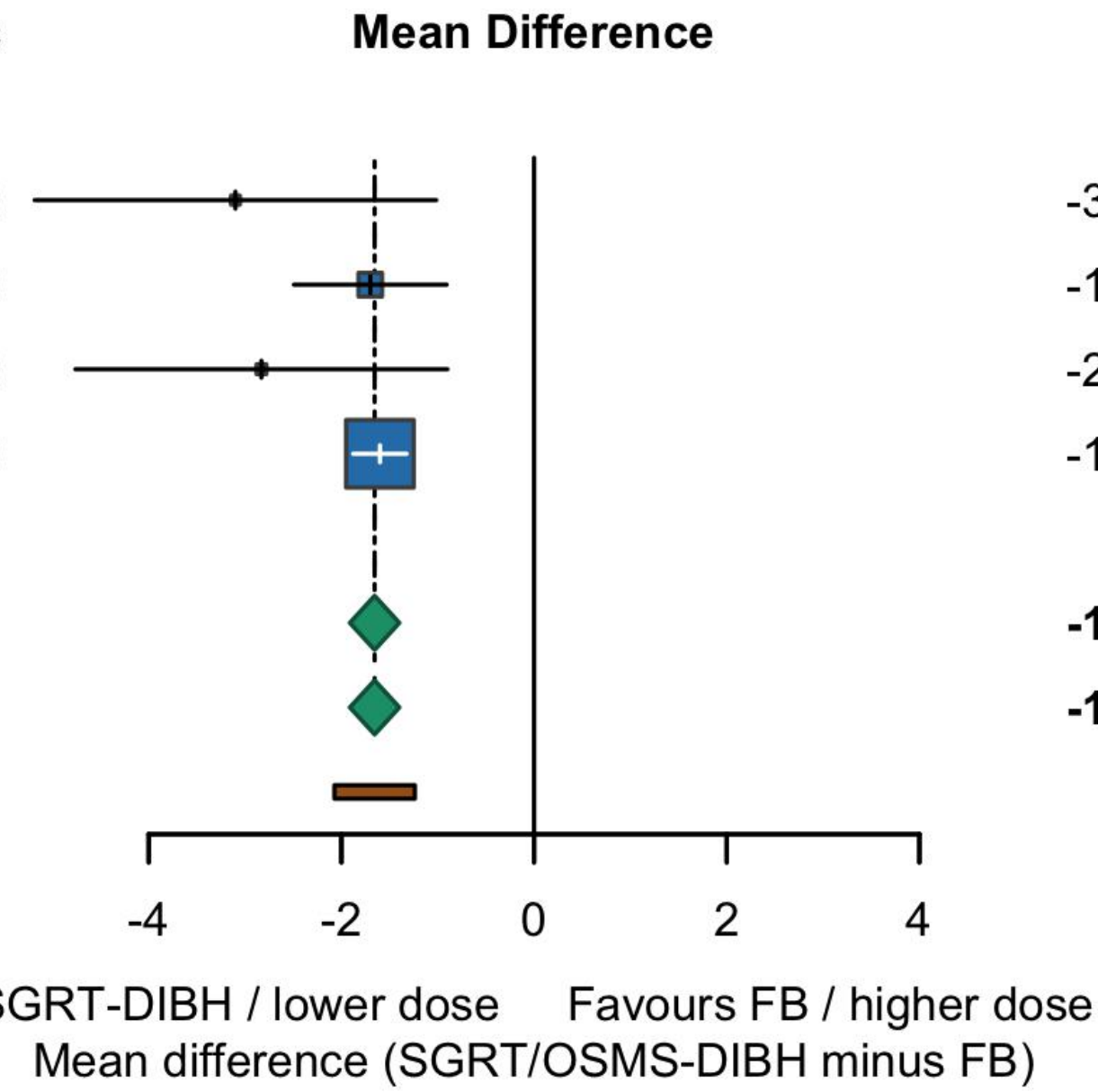

Figure S6.4. Forest plot for Heart V20.

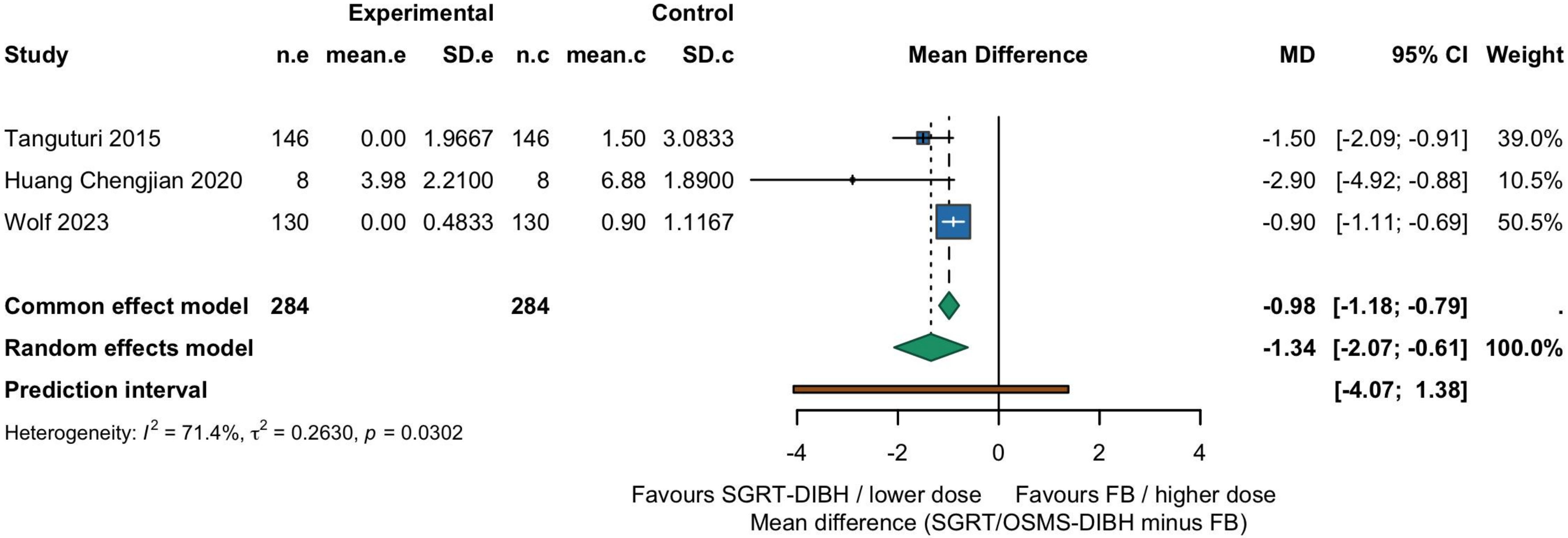

Figure S6.5. Forest plot for Heart V30.

	Experimental			Control					
**Study**	**n.e**	**mean.e**	**SD.e**	**n.c**	**mean.c**	**SD.c**	**MD**	**95% CI**	**Weight**
Eber 2023	10	15.40	15.7000	10	32.00	13.5000	-16.60	[-29.43; -3.77]	9.7%
Kadhim 2024	20	8.90	5.7750	20	17.00	8.0000	-8.10	[-12.42; -3.78]	13.3%
Kügele 2018	20	5.27	11.3475	20	41.72	11.3625	-36.45	[-43.49; -29.41]	12.3%
Rochet 2015	35	18.60	17.5000	35	41.90	17.2000	-23.30	[-31.43; -15.17]	11.8%
Schönecker 2016	9	9.66	6.3000	9	38.55	12.4000	-28.89	[-37.98; -19.80]	11.4%
Schönecker 2024	367	13.90	10.6900	367	26.16	12.8300	-12.26	[-13.97; -10.55]	13.9%
Wolf 2023	130	16.30	8.8500	130	43.40	8.9833	-27.10	[-29.27; -24.93]	13.8%
Zhang 2020	48	9.00	1.9000	48	19.50	5.8000	-10.50	[-12.23; -8.77]	13.8%
**Common effect model**	**639**			**639**			**-15.50**	**[-16.50; -14.49]**	**.**
**Random effects model**							**-20.11**	**[-27.30; -12.93]**	**100.0%**
**Prediction interval**								**[-44.88; 4.65]**	

Heterogeneity: I^2 = 96.7%, τ^2 = 96.2619, p < 0.0001

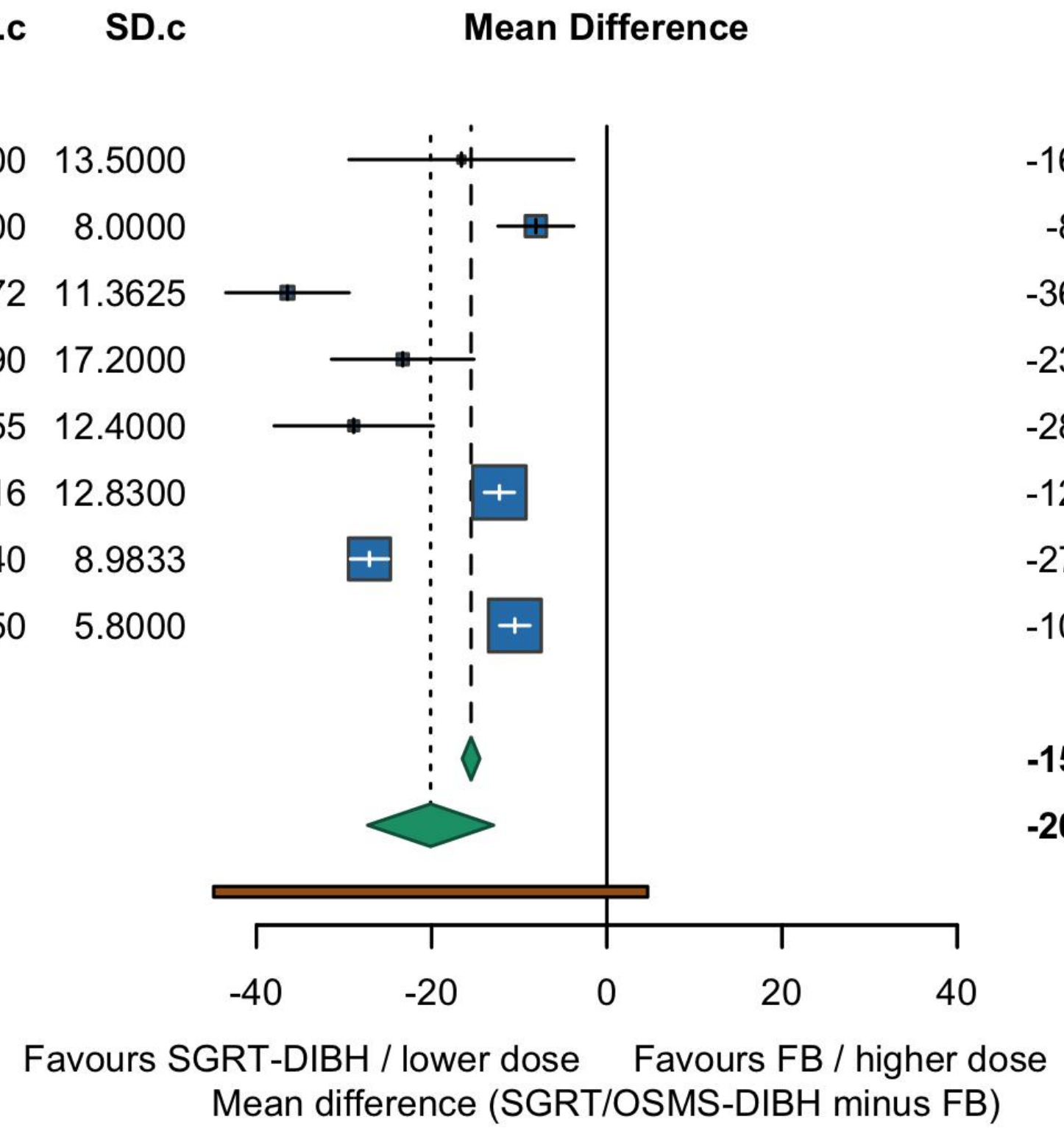

Figure S6.6. Forest plot for LAD Dmax. LAD = left anterior descending coronary artery; Dmax = maximum dose.

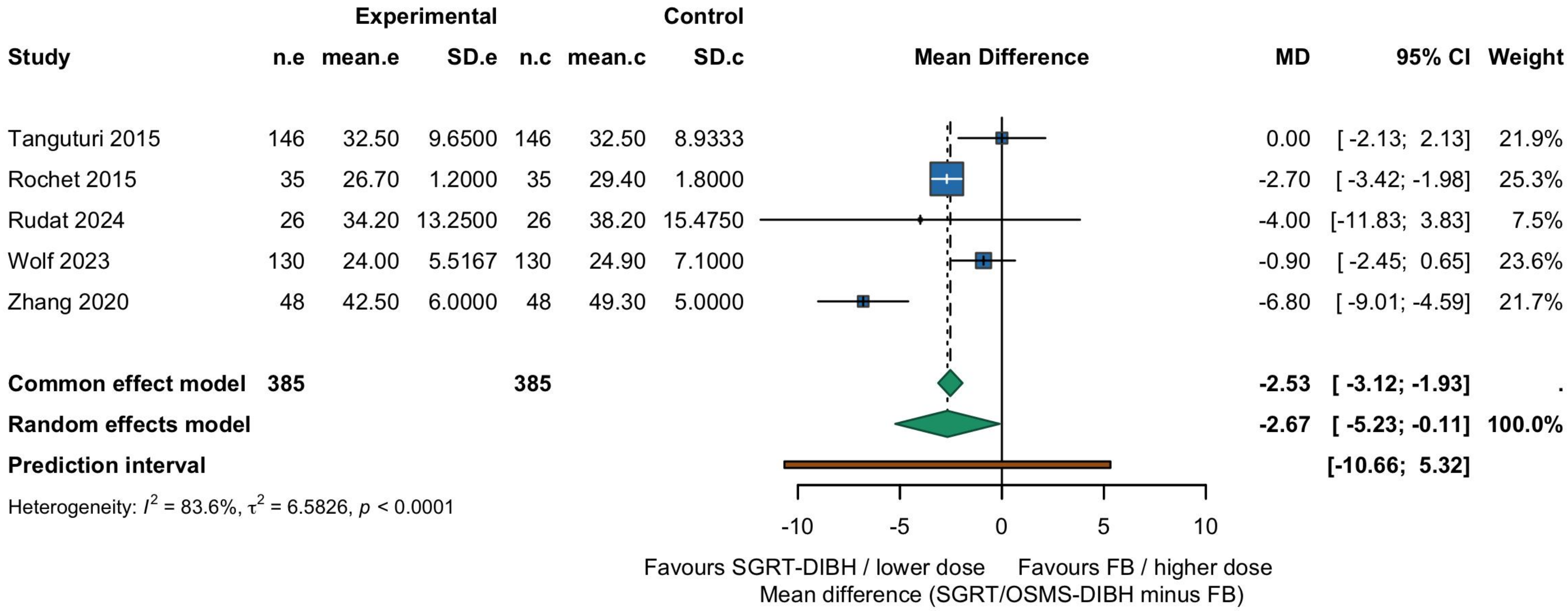

Figure S6.7. Forest plot for ipsilateral lung V5.

Appendix 7. Image-verified setup accuracy: comparative analyses

Forest plots for comparative image-verified residual setup errors are shown below. Effect direction follows the group order reported in the original studies and does not represent a uniform direction of benefit.

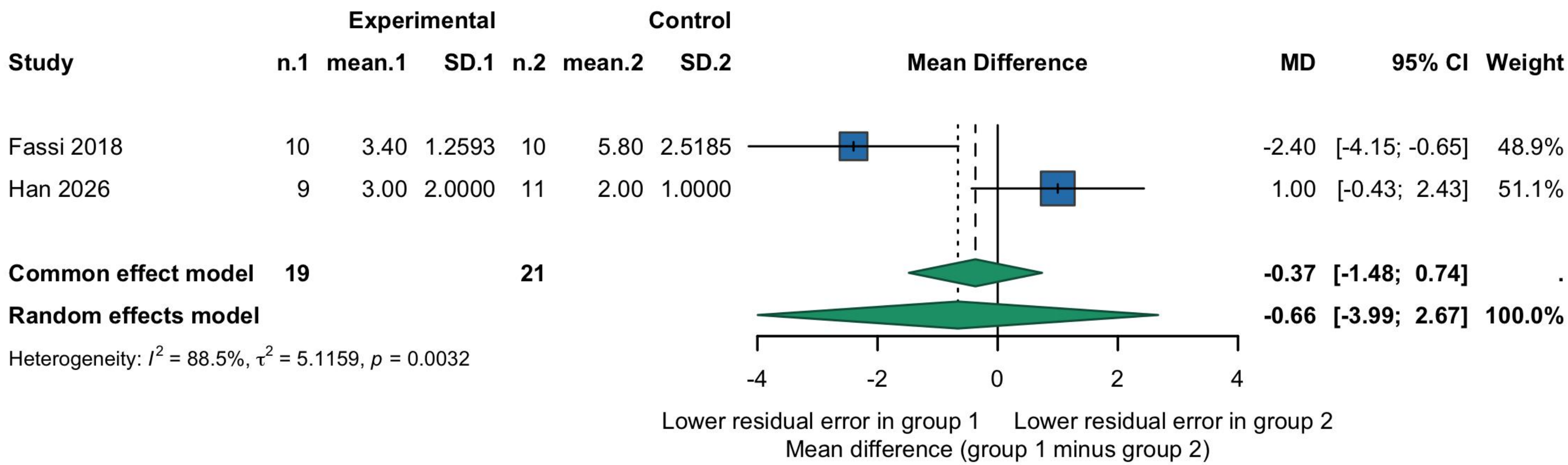

Figure S7.1. Forest plot for comparative 3D vector error.

Study	Experimental n.1	Experimental mean.1	Experimental SD.1	Control n.2	Control mean.2	Control SD.2	MD	95% CI	Weight
Alaimo 2026	288	1.80	1.3576	591	1.94	1.4586	-0.14	[-0.34; 0.06]	9.3%
Bao 2025	150	1.55	1.2500	150	2.09	1.6200	-0.54	[-0.87; -0.21]	8.6%
Chan 2025	20	2.40	1.0918	19	1.90	1.0846	0.50	[-0.18; 1.18]	6.2%
Järvinen 2025	20	0.70	1.4000	25	1.20	1.7000	-0.50	[-1.41; 0.41]	4.8%
Laaksomaa 2019	25	2.00	1.7000	25	1.10	1.9000	0.90	[-0.10; 1.90]	4.4%
Laaksomaa 2022	50	0.50	0.9000	46	0.50	1.0000	0.00	[-0.38; 0.38]	8.3%
Lai 2024	124	2.30	1.7000	124	2.40	1.6000	-0.10	[-0.51; 0.31]	8.1%
Liu 2023	178	1.30	0.9000	193	1.30	1.0000	0.00	[-0.19; 0.19]	9.3%
Lu 2023	10	2.30	2.1578	10	1.80	2.1578	0.50	[-1.39; 2.39]	1.8%
Rossi 2022	51	1.20	1.5000	51	1.70	2.3000	-0.50	[-1.25; 0.25]	5.7%
Rudat 2024	303	0.90	1.8000	303	0.60	0.9000	0.30	[0.07; 0.53]	9.2%
Wu 2023	30	1.50	1.1000	30	1.30	0.8000	0.20	[-0.29; 0.69]	7.5%
Zhang 2020	240	1.28	1.1350	240	1.12	0.6000	0.16	[-0.00; 0.32]	9.4%
Zhao 2023	25	1.10	0.2000	25	2.60	1.3000	-1.50	[-2.02; -0.98]	7.3%
**Common effect model**	**1514**			**1832**			**-0.01**	**[-0.09; 0.07]**	**.**
**Random effects model**							**-0.09**	**[-0.37; 0.19]**	**100.0%**
**Prediction interval**								**[-1.13; 0.94]**	

Heterogeneity: $I^2 = 79.8\%$, $\tau^2 = 0.2106$, $p < 0.0001$

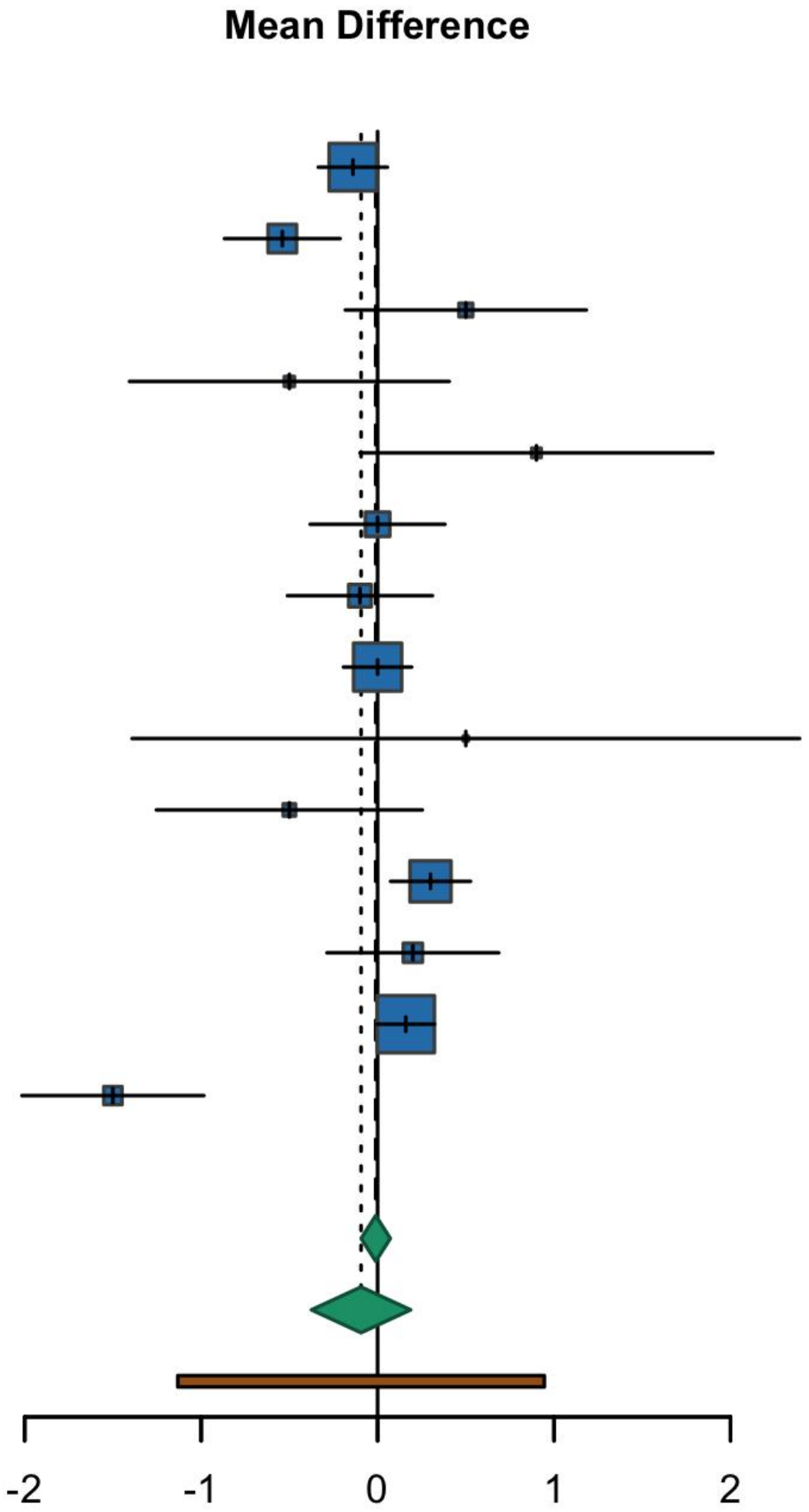

Lower residual error in group 1 Lower residual error in group 2
Mean difference (group 1 minus group 2)

Figure S7.2. Forest plot for comparative LR/LAT residual error. LR/LAT = left-right/lateral.

Study	Experimental n.1	Experimental mean.1	Experimental SD.1	Control n.2	Control mean.2	Control SD.2	MD	95% CI	Weight
Alaimo 2026	288	2.14	1.3576	591	2.64	1.4586	-0.50	[-0.70; -0.30]	8.3%
Bao 2025	150	1.63	1.2100	150	3.37	2.7700	-1.74	[-2.22; -1.26]	7.9%
Chan 2025	20	2.28	1.3962	19	1.31	1.4018	0.97	[0.09; 1.85]	6.8%
Järvinen 2025	20	1.50	1.9000	25	1.70	1.6000	-0.20	[-1.24; 0.84]	6.3%
Laaksomaa 2019	25	1.60	2.3000	25	1.80	2.9000	-0.20	[-1.65; 1.25]	5.1%
Laaksomaa 2022	50	2.30	1.9000	46	1.70	1.9000	0.60	[-0.16; 1.36]	7.2%
Lai 2024	124	2.40	1.7000	124	2.90	1.8000	-0.50	[-0.94; -0.06]	8.0%
Liu 2023	178	1.90	1.4000	193	1.70	1.2000	0.20	[-0.07; 0.47]	8.3%
Lu 2023	10	2.70	2.3798	10	2.40	2.3798	0.30	[-1.79; 2.39]	3.6%
Rossi 2022	51	1.00	1.6000	51	1.50	2.3000	-0.50	[-1.27; 0.27]	7.1%
Rudat 2024	303	1.60	2.4000	303	0.60	1.0000	1.00	[0.71; 1.29]	8.2%
Wu 2023	30	1.70	1.2000	30	1.30	0.9000	0.40	[-0.14; 0.94]	7.8%
Zhang 2020	240	1.69	0.8430	240	1.11	0.8610	0.59	[0.43; 0.74]	8.4%
Zhao 2023	25	1.20	0.2000	25	3.60	2.1000	-2.40	[-3.23; -1.57]	7.0%
**Common effect model**	**1514**			**1832**			**0.14**	**[0.04; 0.23]**	**.**
**Random effects model**							**-0.15**	**[-0.67; 0.38]**	**100.0%**
**Prediction interval**								**[-2.23; 1.94]**	

Heterogeneity: $I^2 = 94.1\%$, $\tau^2 = 0.8579$, $p < 0.0001$

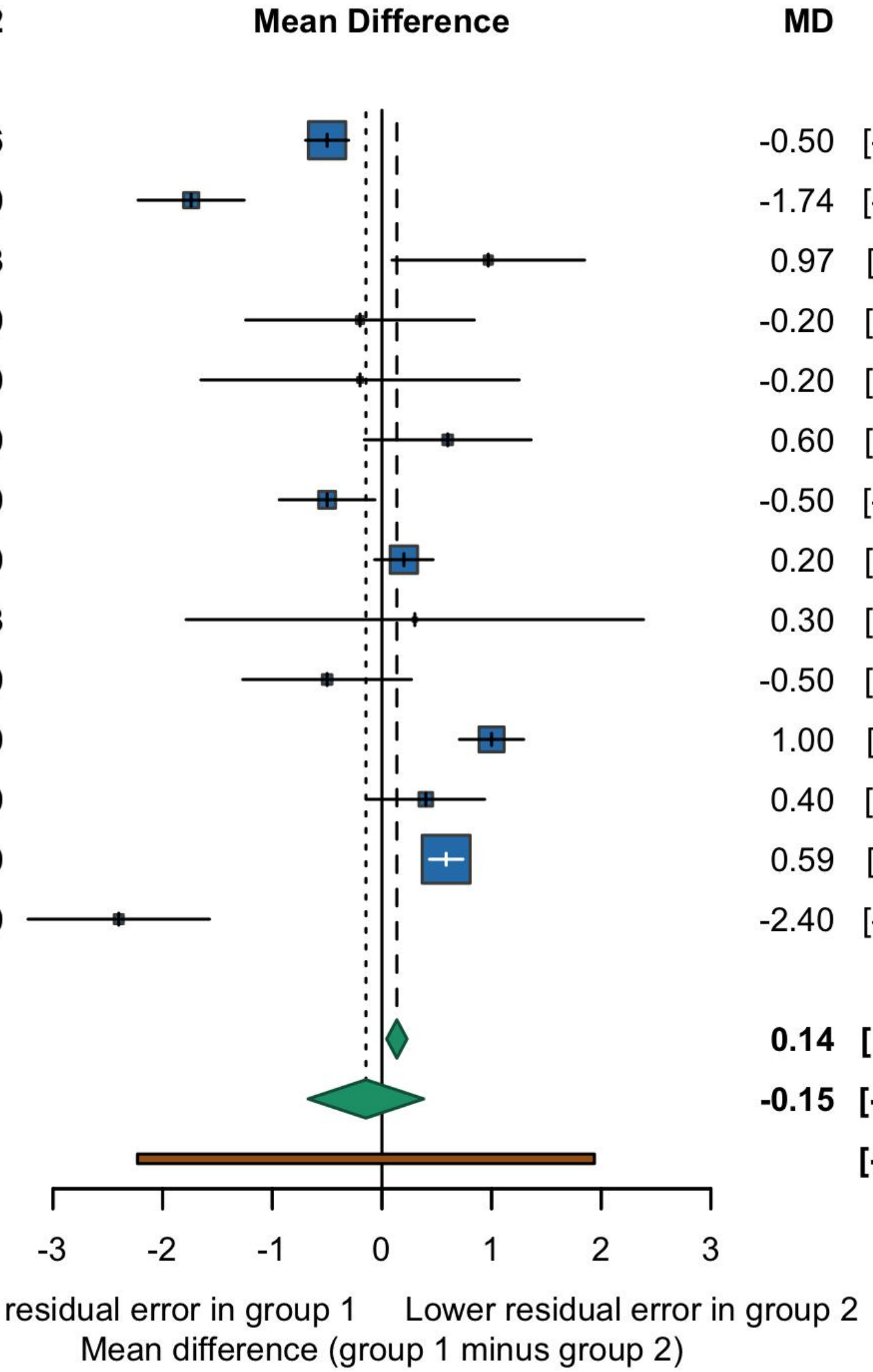

Figure S7.3. Forest plot for comparative SI/LNG/CC residual error. SI/LNG/CC = superior-inferior/longitudinal/craniocaudal.

	Experimental			Control					
**Study**	**n.1**	**mean.1**	**SD.1**	**n.2**	**mean.2**	**SD.2**	**MD**	**95% CI**	**Weight**
Alaimo 2026	288	2.13	1.3576	591	2.52	1.4586	-0.39	[-0.59; -0.19]	7.8%
Bao 2025	150	1.79	1.5300	150	3.34	2.6900	-1.55	[-2.05; -1.05]	7.1%
Byrne 2024	14	0.40	0.7000	12	0.40	0.7000	0.00	[-0.54; 0.54]	6.9%
Chan 2025	20	1.86	1.0603	19	1.88	1.0641	-0.02	[-0.69; 0.65]	6.5%
Järvinen 2025	20	0.80	1.4000	25	1.60	1.6000	-0.80	[-1.68; 0.08]	5.7%
Laaksomaa 2019	25	1.10	2.1000	25	0.60	1.8000	0.50	[-0.58; 1.58]	4.9%
Laaksomaa 2022	50	0.70	1.1000	46	0.70	1.2000	0.00	[-0.46; 0.46]	7.2%
Lai 2024	124	2.30	1.8000	124	2.80	2.1000	-0.50	[-0.99; -0.01]	7.1%
Liu 2023	178	1.70	1.1000	193	1.60	1.1000	0.10	[-0.12; 0.32]	7.8%
Lu 2023	10	2.10	2.1112	10	2.60	2.1112	-0.50	[-2.35; 1.35]	2.8%
Rossi 2022	51	1.40	1.5000	51	1.50	1.6000	-0.10	[-0.70; 0.50]	6.7%
Rudat 2024	303	1.30	1.6000	303	0.40	0.6000	0.90	[0.71; 1.09]	7.9%
Wu 2023	30	2.00	1.4000	30	2.00	1.1000	0.00	[-0.64; 0.64]	6.6%
Zhang 2020	240	1.42	0.8880	240	0.94	0.8270	0.48	[0.32; 0.63]	7.9%
Zhao 2023	25	1.20	0.2000	25	3.00	1.3000	-1.80	[-2.32; -1.28]	7.0%
**Common effect model**	**1528**			**1844**			**0.14**	**[0.06; 0.22]**	**.**
**Random effects model**							**-0.23**	**[-0.62; 0.16]**	**100.0%**
**Prediction interval**								**[-1.79; 1.33]**	

Heterogeneity: $I^2 = 93.6\%$, $\tau^2 = 0.4896$, $p < 0.0001$

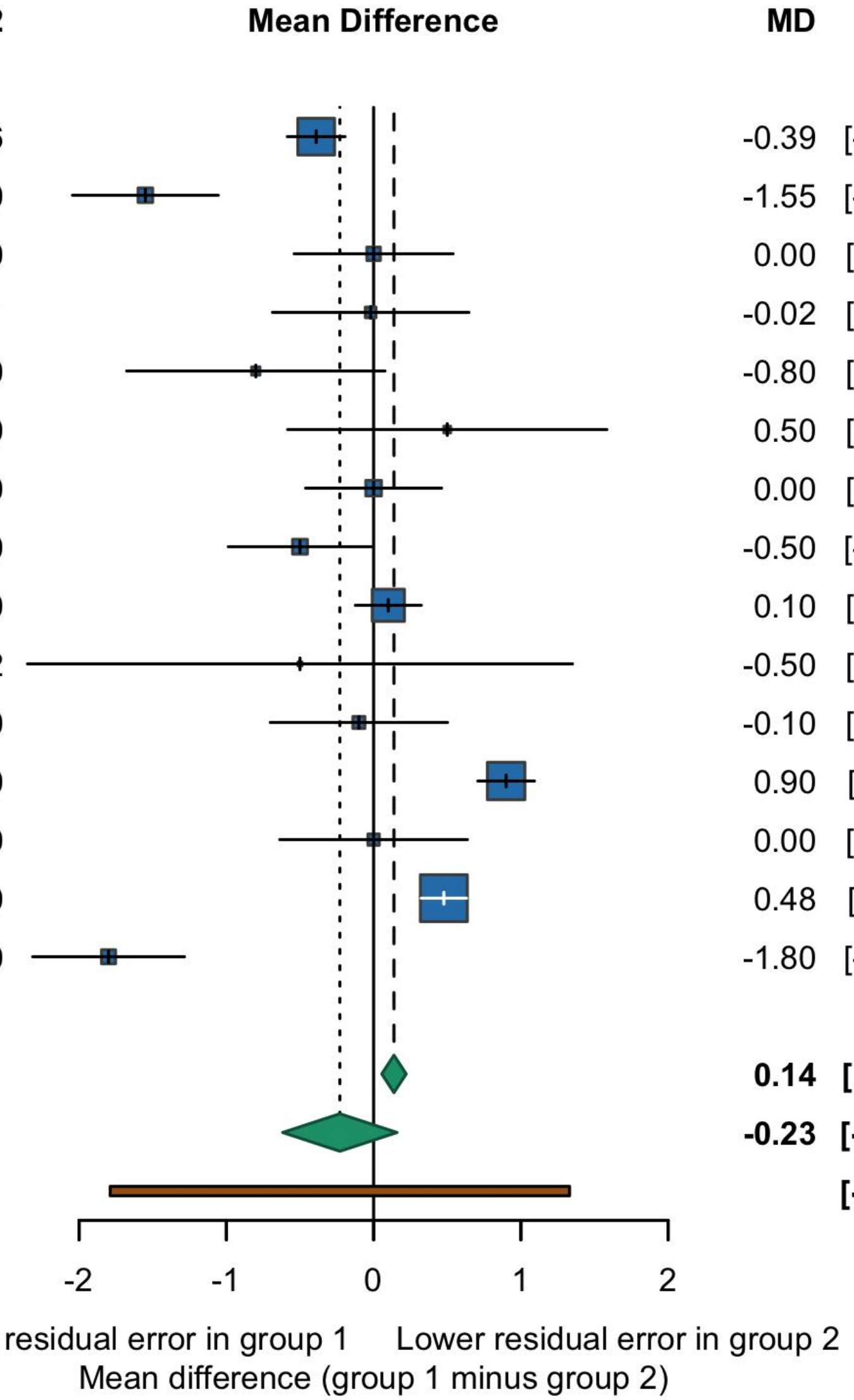

Figure S7.4. Forest plot for comparative AP/VRT residual error. AP/VRT = anterior-posterior/vertical.

Appendix 8. Image-verified setup accuracy: single-arm analyses

Table S8.1. Single-arm pooled residual setup-error estimates.

Outcome	k	Total n	Pooled mean (mm)	95% CI	I2 (%)
3D vector error	2	745	6.21	4.19 to 8.22	97.0
LR/LAT error	7	265	0.88	0.47 to 1.29	80.9
SI/LNG/CC error	7	265	2.02	1.40 to 2.64	84.9
AP/VRT error	7	265	1.13	0.82 to 1.44	68.8

Note. Single-arm pooled means describe the overall magnitude of image-verified setup error under SGRT/OSMS-assisted DIBH and are not comparative effects. The primary clinical interpretation focuses on positioning discrepancies observed by image verification after SGRT-guided setup; study-specific pre-/post-correction measurement timing is reported in Table S9.1, and values explicitly reported as post-correction residual discrepancies are not interpreted in the same way as initial setup error. The pooled 3D-vector estimate is based on only two studies in the primary analysis ($I^2 = 97.0\%$). A 3D vector is the magnitude of displacement synthesized across multiple orthogonal directions and is defined differently from single-axis LR/LAT, SI/LNG/CC, or AP/VRT errors; its numerical value is therefore generally larger than a single-axis setup error.

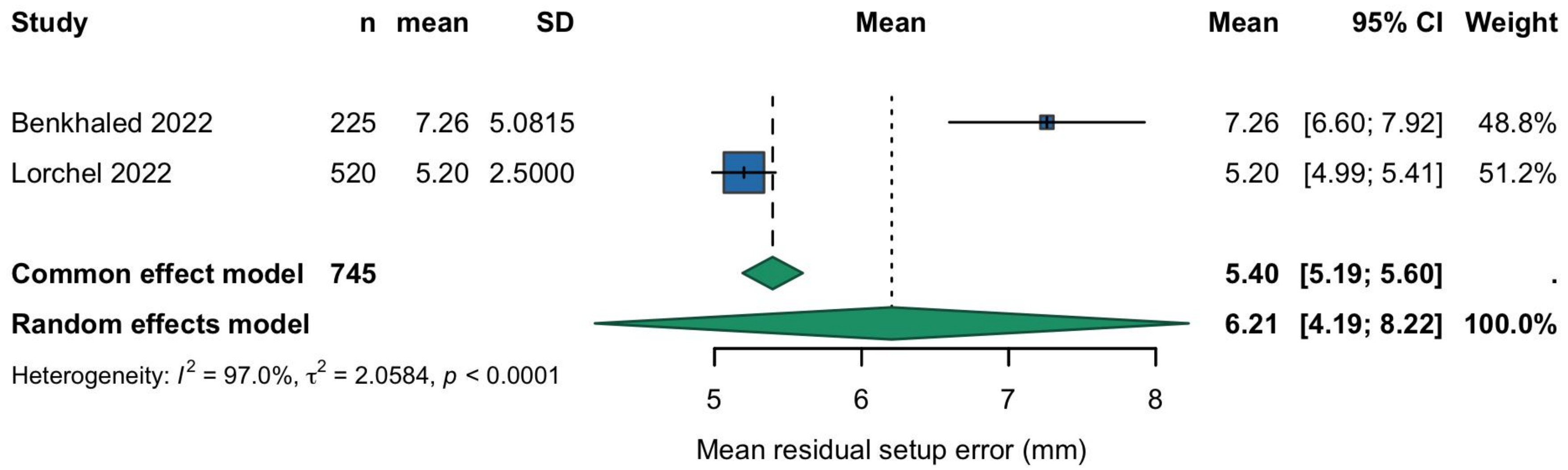

Figure S8.1. Forest plot for single-arm 3D vector error.

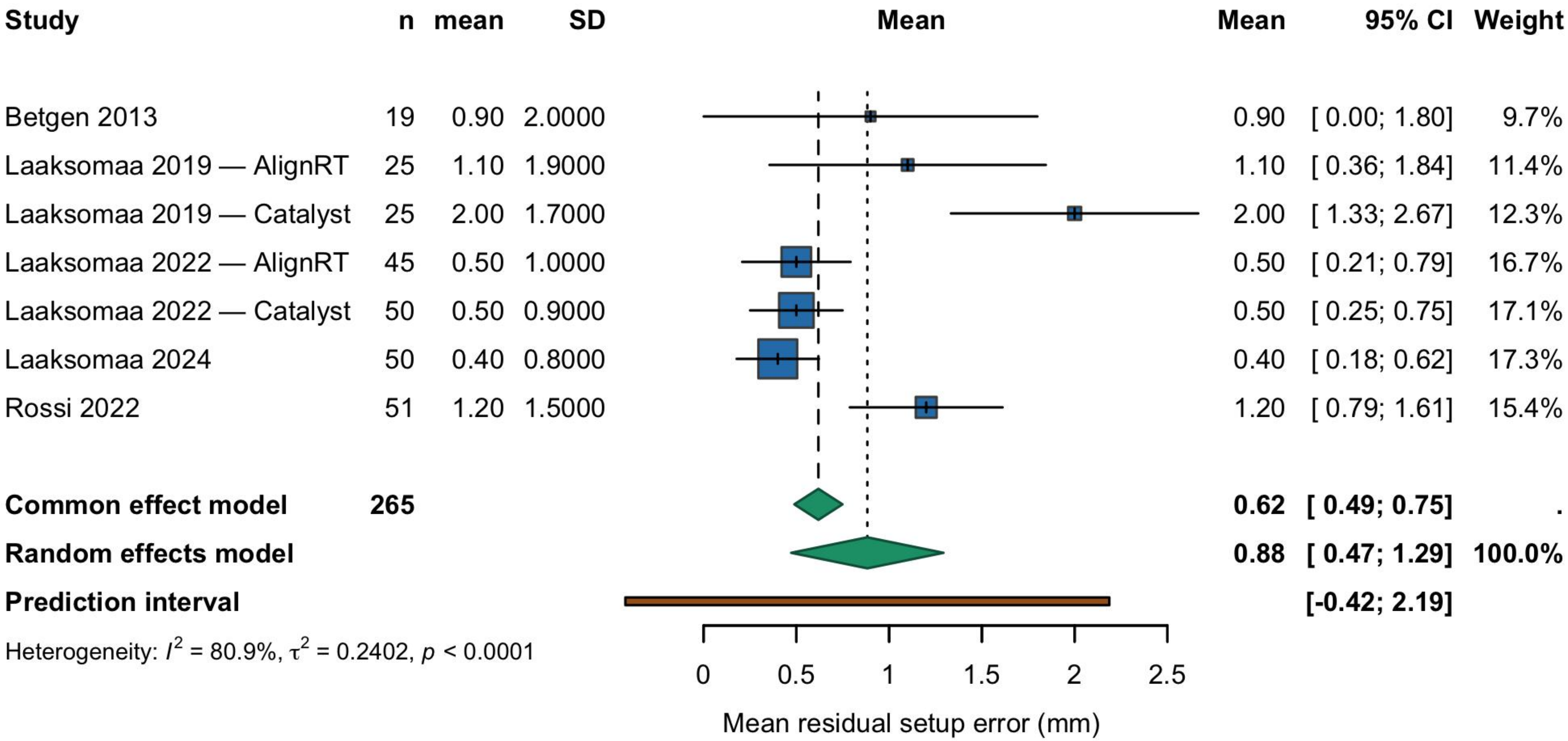

Figure S8.2. Forest plot for single-arm LR/LAT residual error. LR/LAT = left-right/lateral.

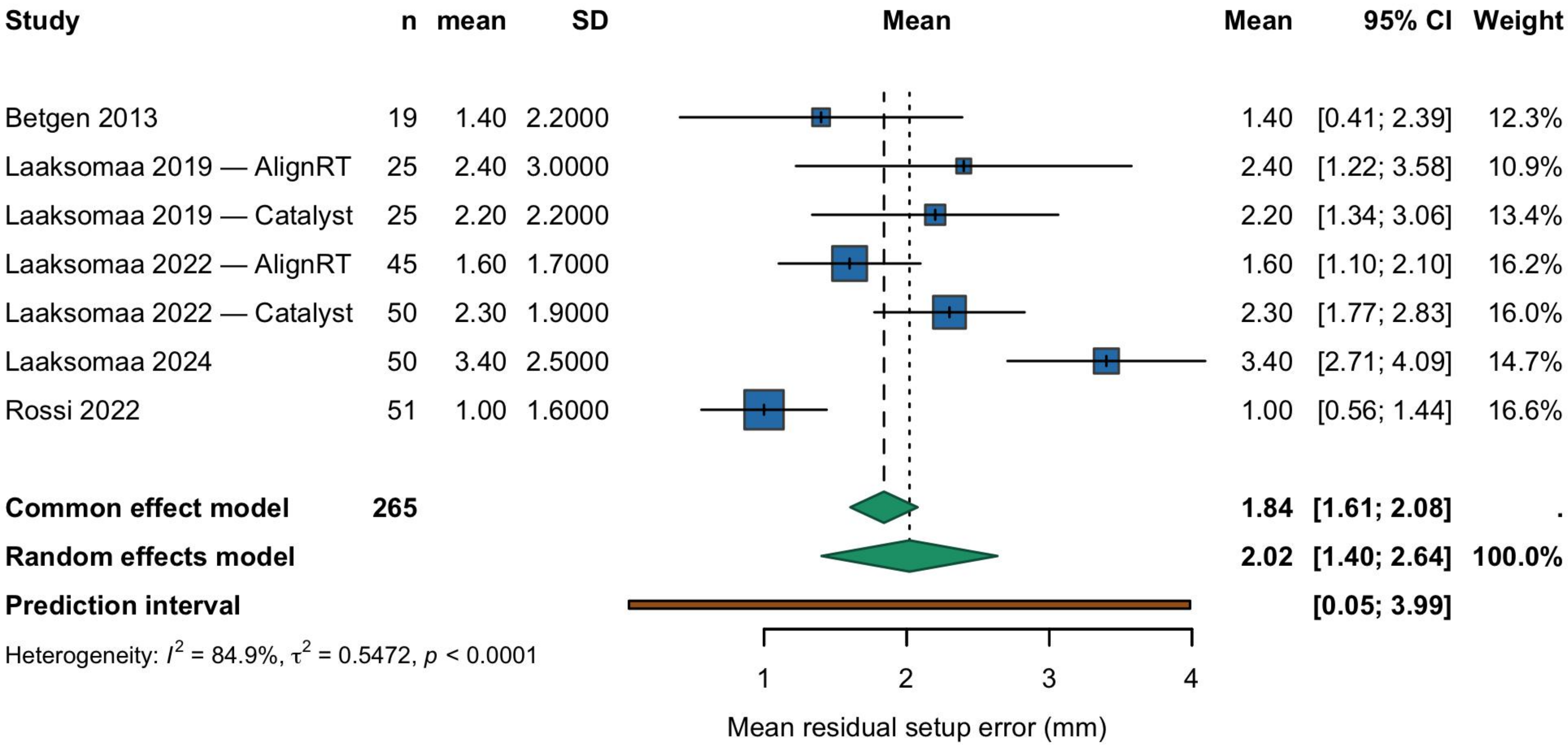

Figure S8.3. Forest plot for single-arm SI/LNG/CC residual error. SI/LNG/CC = superior-inferior/longitudinal/craniocaudal.

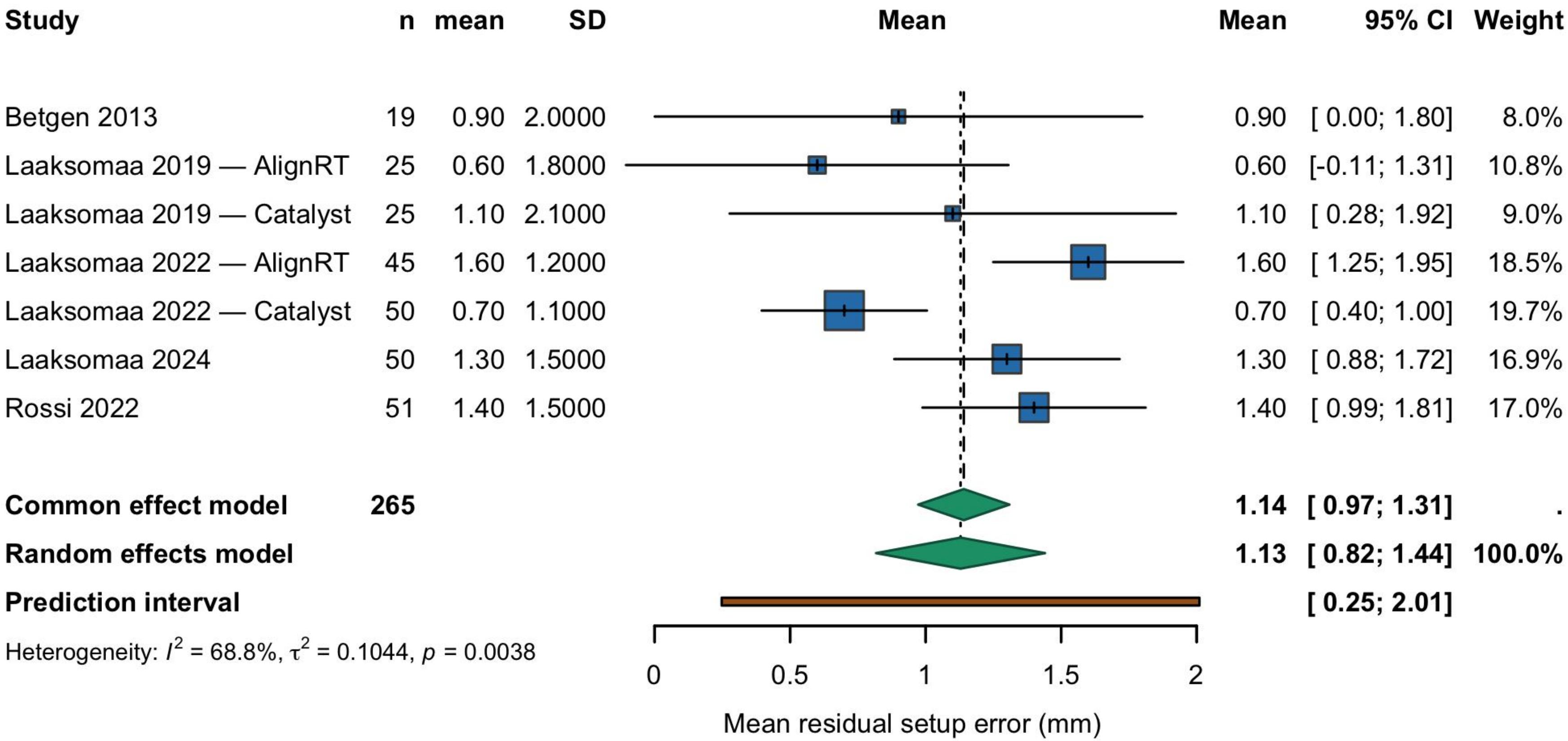

Figure S8.4. Forest plot for single-arm AP/VRT residual error. AP/VRT = anterior-posterior/vertical.

Appendix 9. SGRT/OSMS technical heterogeneity, dosimetric definitions, and exploratory SGRT-system-stratified analyses

This appendix summarizes, study by study, SGRT/OSMS implementation details, image-verification and quality-assurance information, differences in dosimetric definitions, and exploratory SGRT-system-stratified results for outcomes meeting the minimum study-count requirement. Information not reported in the primary studies is coded as NR and is not inferred.

Table S9.1. SGRT/OSMS workflow, image-verification, and technical characteristics of the 52 included studies.

Study	SGRT/OSMS system/configuration	Reference surface/ROI	Gating/action threshold	Imaging-verification modality	Error-measurement timing	Imaging frequency/number of fractions	Reference-surface update after IGRT	Calibration/QA	Treatment-couch position/angle; gantry-angle/FOV occlusion
Zhang 2023	OSMS/AlignRT-DIBH	external skin over the treatment region; unstable axillary area avoided	3 mm / 3°	image verification per study workflow	per source-study workflow	NR	NR	NR	NR
Zhang 2020	OSMS/Catalyst-DIBH	OSMS reference surface/respiratory monitoring region	~5-mm gating window; reproducibility controlled to ~3 mm	NR	NR	NR	NR	NR	beam held when outside the window; visual feedback provided
Abdollahi 2022	Catalyst/Sentinel-DIBH	Sentinel simulation reference; Catalyst treatment-room surface	3-mm gating window	NR	NR	NR	NR	NR	Sentinel used at CT simulation and Catalyst at treatment; audiovisual feedback provided
Schönecker 2016	Catalyst/Sentinel-DIBH	Sentinel/Catalyst reference surface	4 mm, progressively tightened to 2 mm	NR	NR	NR	NR	NR	visual feedback provided
Huang Chengjian 2020	OSMS-DIBH	NR	NR	NR	NR	NR	NR	NR	NR
Wolf 2023	Sentinel/Catalyst-DIBH	NR	5-mm gating window	NR	NR	NR	NR	NR	NR
Kügele 2018	Catalyst-DIBH	~2-cm-diameter ROI near the xiphoid; baseline/new DIBH reference surface rebuilt each fraction	3-mm gating window	image verification per source-study workflow	after SGRT setup / study-specific time point	each fraction	baseline/reference surface re-established	NR	non-rigid tracking; residual setup error not transferred
Mkanna 2018	AlignRT-DIBH	AlignRT surface	NR	portal imaging/CBCT (as needed)	after SGRT setup	as needed	NR	NR	NR
Tanguturi 2015	AlignRT-DIBH	chest-wall ROI	±3 mm	NR	NR	NR	NR	NR	three-camera AlignRT
Wu 2023a	Sentinel-DIBH	virtual gating point 1-2 cm above the xiphoid	3-mm gating window	NR	NR	NR	NR	NR	NR
Schönecker 2024	Catalyst-DIBH	Sentinel simulation reference/Catalyst treatment-room reference	4 mm, can be tightened to 2 mm	NR	NR	NR	NR	NR	NR
Rudat 2024a	SGRT-DIBH	AlignRT surface	NR	CBCT	image-verified	CBCT	NR	NR	SGRT-only versus

**Study**	**SGRT/OSMS system/configuration**	**Reference surface/ROI**	**Gating/action threshold**	**Imaging-verification modality**	**Error-measurement timing**	**Imaging frequency/number of fractions**	**Reference-surface update after IGRT**	**Calibration/QA**	Treatment-couch position/angle; gantry-angle/FOV occlusion
					residual error	verification performed in the study			CBCT workflow comparison
Kadhim 2024	Catalyst HD SGRT-DIBH	~4-cm respiratory ROI; VitalHold/Catalyst HD	NR	NR	NR	NR	NR	NR	inner ring-gantry camera may be occluded, with primary reliance on the main camera; patient habitus/gantry geometry affects surface coverage
Zeng 2023	SGRT/RPM-DIBH	chest ROI; abdominal ROI/surrogate signal required when abdominal-pattern DIBH is identified	±3 mm / ±3°	NR	NR	NR	NR	NR	a chest ROI may miss abdominal-pattern DIBH; discrimination is limited when VCE ≤5 mm
Li 2021	ABC+Sentinel-DIBH	Sentinel surface monitoring	~3-mm surface-amplitude alert	NR	NR	NR	NR	NR	surface amplitude may still vary by >3 mm despite stable ABC volume
Eber 2023	AlignRT-DIBH	NR	NR	NR	NR	NR	NR	NR	NR
Vuong 2018	AlignRT-DIBH	AlignRT surface	0.3 cm	Portal imaging	verification after setup/correction	weekly	reference surface can be reacquired after correction	NR	three-camera pods
Rochet 2015	AlignRT-DIBH	NR	NR	NR	NR	NR	NR	NR	NR
Lu 2023a	SGRT-DIBH	ROI encompassing the entire ipsilateral breast with extension to surrounding surface	±0.3 cm / ±3°	MV or orthogonal kV	after SGRT and before image correction; final couch shift determined by imaging	weekly MV for tangential fields; daily kV for VMAT	NR	NR	NR
Rossi 2022	SGRT-DIBH	SGRT surface	NR	orthogonal imaging/IGRT	after SGRT setup and after image-guided correction	per study IGRT workflow	NR	NR	study indicated that imaging should not be omitted without reassessing margins
Penninkhof 2022	AlignRT-DIBH	SGRT surface	NR	image guidance	before and after image-guided correction	daily	NR	NR	emphasized daily imaging and correction
Laaksomaa 2022	AlignRT/Catalyst-DIBH	AlignRT 6D reference surface; Catalyst BH reference surface established each fraction	system-specific	orthogonal IGRT	after SGRT setup	daily recommended	NR	NR	differences among systems/workflows
Lu 2023b	AlignRT OSI + 2DkV/CBCT-DIBH	OSI surface	NR	2D kV + CBCT (CBCT as reference)	CBCT assessment after OSI/2D kV	daily 2D kV; CBCT used as reference	NR	NR	limited agreement between OSI and CBCT; daily 2D kV

Study	SGRT/OSMS system/configuration	Reference surface/ROI	Gating/action threshold	Imaging-verification modality	Error-measurement timing	Imaging frequency/number of fractions	Reference-surface update after IGRT	Calibration/QA	Treatment-couch position/angle; gantry-angle/FOV occlusion
									could not be omitted
Hamming 2019	AlignRT+ABC-DIBH	AlignRT 3D surface	NR	CBCT	after surface-guided setup	study workflow	NR	NR	surface imaging can identify displacements not detected by ABC
Laaksomaa 2019	AlignRT/Catalyst-DIBH	AlignRT/Catalyst surface	~3-mm range	IGRT	after SGRT setup / after IGRT	study workflow	NR	NR	IGRT can further reduce residual error
Fassi 2018	Optical tracking-DIBH	NR	NR	NR	NR	NR	NR	NR	NR
Tang 2014	AlignRT-DIBH	AlignRT surface	3 mm / 3°	portal films	after SGRT setup	weekly	NR	NR	three cameras
Abubakar 2023	ToF optical surface camera + CBCT/EPID-DIBH	ToF optical surface	NR	CBCT + EPID	surface monitoring compared with imaging	study workflow	NR	NR	gantry angle may introduce depth error
Wu 2023b	OSMS/Catalyst-DIBH	CT-side AlignRT or Sentinel/Catalyst reference	NR	CBCT	after first-fraction setup	first fraction	NR	NR	comparison with versus without CT-side optical equipment
Dekker 2023	SGRT-DIBH	Varian Identify surface	NR	CBCT	after SGRT setup	study workflow	NR	NR	NR
Benkhaled 2022	vDIBH + SGRT tracking + daily CBCT	SGRT surface	NR	CBCT	after surface monitoring	daily	NR	NR	interfraction heart-position variation observed
Rong 2014	AlignRT+RPM-DIBH	AlignRT surface	vertical ±3 mm; displacement magnitude 5 mm	NR	NR	NR	NR	NR	external-to-target correlation compared with RPM
Alaimo 2026	AlignRT Advance-DIBH	AlignRT Advance	NR	NR	NR	NR	NR	NR	NR
Nankali 2022	AlignRT/RPM-DIBH	AlignRT surface	NR	internal chest-wall/imaging reference	surface compared with internal chest wall	study workflow	NR	NR	external-internal offset >2 mm in ~13% of fractions
Byrne 2024	Optical surface tracking/RPM-DIBH	chest surface or abdominal block	NR	NR	NR	NR	NR	NR	chest surface correlated more closely with internal anatomy than the abdominal block
Zhao 2023	Varian Identify SGRT-DIBH	Varian Identify surface	±0.3 cm / ±3°	Portal imaging	after SGRT setup	weekly	reference surface reacquired when necessary	commissioning/QA consistent with TG-302-related procedures	reference surface reacquired in 7 cases
Lorchel 2022	AlignRT InBore-DIBH	setup ROI included treated breast/supraclavicular	2 mm vertically; 3 mm/3° in other directions	CBCT	online verification after SGRT setup	online CBCT	NR	NR	narrower InBore field of view limits the monitoring ROI

Study	SGRT/OSMS system/configuration	Reference surface/ROI	Gating/action threshold	Imaging-verification modality	Error-measurement timing	Imaging frequency/number of fractions	Reference-surface update after IGRT	Calibration/QA	Treatment-couch position/angle; gantry-angle/FOV occlusion
		region/sternum/contralateral side; InBore monitoring ROI mainly covered the treated breast							
Parsons 2023	VisionRT SGRT/ABC-DIBH	VisionRT surface	NR	NR	NR	NR	NR	NR	breath-hold stability/reproducibility compared with an ABC workflow
Delombaerde 2021	SDX + intra-bore surface tracking-DIBH	intra-bore surface	NR	portal images	during irradiation/intrafraction monitoring	study workflow	NR	NR	closed-bore; manual beam interruption required
Alderliesten 2013	AlignRT-DIBH	AlignRT central camera; two ROIs compared	NR	CBCT	after surface-guided setup; CBCT bony/heart registration	online CBCT	NR	NR	moderate correlation between surface and heart position
Betgen 2013	AlignRT-DIBH	AlignRT central camera	NR	online IGRT	after surface-guided setup	online	NR	daily QA; recalibrated when RMS >1 mm	substantial interfraction variation; online image correction required
Guo 2026	SGRT/RPM-DIBH	sternum + partial left-breast ROI	~3 mm	CBCT/image verification	residual 6D error after SGRT	study workflow	NR	NR	potentially affected by camera occlusion, couch movement, and residual vertical error
Han 2026	ExacTrac Dynamic SGRT-DIBH	ExacTrac Dynamic optical/thermal imaging	system-specific	stereoscopic X-ray	X-ray verification after surface positioning	study workflow	NR	vendor performance/QA as reported in the study	integrated 6DOF; optical/thermal imaging combined with X-ray
Järvinen 2025	Sentinel/Catalyst-DIBH	Sentinel xiphoid point; Catalyst at treatment	3-mm vertical gating; SGRT tolerance tightened from 8/12 mm to 5 mm	image guidance	after SGRT setup	study workflow	NR	NR	modified immobilization and tighter tolerances improved residual error
Chan 2025	SGRT/ABC/vDIBH	AlignRT surface	3 mm / 3°	CBCT	after SGRT setup with online correction	weekly	NR	NR	NR
Lai 2024	Sentinel/Catalyst SGRT-DIBH	Sentinel at simulation/Catalyst at treatment; relatively broad ROI	1.5-3-mm gating window	CBCT/image verification	after SGRT setup	study workflow	NR	NR	NR
Rudat 2024b	AlignRT-DIBH	three-camera AlignRT; breast/chest-wall ROI	NR	CBCT	after SGRT setup	study workflow	NR	NR	SGRT-only margins 4/6/4 mm; intrafraction contribution ≤1 mm

Study	SGRT/OSMS system/configuration	Reference surface/ROI	Gating/action threshold	Imaging-verification modality	Error-measurement timing	Imaging frequency/number of fractions	Reference-surface update after IGRT	Calibration/QA	Treatment-couch position/angle; gantry-angle/FOV occlusion
Buschmann 2024	ExacTrac Dynamic thermo-optical SGRT-DIBH	ExacTrac Dynamic thermo-optical surface	NR	stereoscopic X-ray + CBCT	surface compared with X-ray/CBCT	study workflow	NR	NR	ceiling-mounted thermo-optical camera FOV was not occluded by the gantry at the evaluated gantry angles; moderate surface-internal correlation
Laaksomaa 2024	AlignRT/Catalyst-DIBH	multicenter AlignRT/Catalyst	center-/system-specific	IGRT	after SGRT setup	daily	NR	center-specific QA workflows	equipment, immobilization, and tolerances differed across centers
Bao 2025	Sentinel/Catalyst +CBCT-DIBH	Sentinel/Catalyst surface	NR	pre-treatment and intrafraction CBCT	pre-treatment and intrafraction	pre-treatment + intrafraction	NR	NR	discrepancy decreased after intrafraction CBCT correction
Crop 2023	Catalyst/Sentinel-DIBH	Halcyon closed-bore; three anterior cameras, with the central camera aligned to the true isocenter	NR	CBCT	after SGRT positioning / during treatment workflow	study workflow	NR	daily ~1-mm QA	closed-bore; patient habitus may reduce tracking accuracy at the true isocenter; no automatic gating, requiring manual beam interruption
Liu 2023	C-RAD optical body surface-DIBH	C-RAD optical surface tracking	NR	CBCT	after surface-guided setup	study workflow	NR	NR	compared with ABC; shorter irradiation time in the optical-surface group

Note. NR = not reported in the primary study or not reliably determinable from the full text. Image-verified setup error was defined as the geometric positioning discrepancy identified by image verification relative to the planned or image-based reference position after SGRT/OSMS-guided surface positioning. Because image-verification and correction workflows were not fully uniform across the included studies, this outcome was extracted according to the measurement timing and definition reported in each primary study. Table S9.1 records, for each study, the imaging-verification modality, timing of error measurement, verification frequency/number of fractions, and whether the reference surface was updated after IGRT correction; unreported information is coded as NR. SGRT = surface-guided radiotherapy; OSMS = optical surface monitoring system; IGRT = image-guided radiotherapy.

Table S9.2. Definitions and meta-analysis handling for studies reporting primary dosimetric outcomes.

Study	Heart definition	LAD definition	Lung V20 volume	Meta-analysis handling
Zhang 2023	NR	NR	left/ipsilateral lung	Included
Zhang 2020	NR	NR	left/ipsilateral lung	Included
Abdollahi 2022	RTOG	delineated; length NR	ipsilateral lung	Included
Schönecker 2016	Feng atlas	Feng atlas	ipsilateral lung	Included
Huang Chengjian 2020	institutional definition (atlas NR)	NR	left/ipsilateral lung	Included
Wolf 2023	RTOG/ESTRO/Feng	source-study extent	bilateral lungs; only ipsilateral-lung	ipsilateral-lung outcome

Study	Heart definition	LAD definition	Lung V20 volume	Meta-analysis handling
			result included	included
Kügele 2018	myocardium and great vessels to coronary origins	visible extent; ≈6 mm	ipsilateral lung	Included
Mkanna 2018	to pulmonary artery bifurcation; RTOG 1304/B51	—	ipsilateral lung	Included
Tanguturi 2015	RTOG consensus contour	—	lung V20 (paired)	Included
Schönecker 2024	Feng atlas	Feng atlas	ipsilateral lung	Included
Rudat 2024a	NR	NR	NR	Included
Kadhim 2024	NR	NR	NR	Included
Zeng 2023	NR	NR	NR	Included
Li 2021	institutional definition (atlas NR)	LAD not used as a poolable primary outcome	whole-lung V20	whole-lung V20 excluded
Eber 2023	Feng atlas	Feng atlas	—	Included
Vuong 2018	NR	NR	NR	Included
Rochet 2015	RTOG/Michigan/Feng	origin to apex; ≈5 mm	left lung	Included
Tang 2014	RTOG	—	NR	only anatomically equivalent outcomes included
Rong 2014	RTOG	NR	NR	only anatomically equivalent outcomes included
Guo 2026	Feng-related	extent NR	left lung	Included
Han 2026	ESTRO-related	length NR	left lung	non-FB comparator; excluded
Chan 2025	planning CT/CBCT	—	—	non-FB comparator; excluded

Note. Only studies reporting at least one prespecified primary dosimetric outcome or a related comparable dose metric are shown; studies contributing setup outcomes only are not listed. NR = not reported; — = not applicable/not assessed. The primary meta-analysis included only prespecified outcomes with anatomically equivalent definitions and an FB comparator. Whole-/bilateral-lung V20 was not pooled with ipsilateral-lung V20; an unreported contour definition alone was not a reason for exclusion. Original CT datasets were not uniformly recontoured. FB = free breathing; LAD = left anterior descending coronary artery.

Table S9.3. Exploratory SGRT-system-stratified analyses meeting the minimum study-count criterion.

Outcome	SGRT-system stratum	k	MD (95% CI)	Interpretation
Heart Dmean	AlignRT/VisionRT	3	-1.00 Gy (-1.19 to -0.81)	Dose-reduction direction
Heart Dmean	Catalyst/C-RAD	5	-1.04 Gy (-1.57 to -0.51)	Dose-reduction direction
Heart Dmean	OSMS	4	-1.72 Gy (-2.02 to -1.43)	Dose-reduction direction
Heart Dmean	Other SGRT/OSMS	6	-1.02 Gy (-1.47 to -0.57)	Dose-reduction direction
Ipsilateral lung V20	Catalyst/C-RAD	3	-3.46 percentage points (-6.90 to -0.02)	Dose-reduction direction
Ipsilateral lung V20	OSMS	4	-2.98 percentage points (-3.64 to -2.32)	Dose-reduction direction
Ipsilateral lung V20	Other SGRT/OSMS	5	-1.06 percentage points (-2.03 to -0.08)	Dose-reduction direction

Note. These SGRT-system-stratified analyses are exploratory and are reported only when each system subgroup contains at least three comparable independent studies. LAD Dmean and ipsilateral lung Dmean did not form multiple interpretable system subgroups meeting the minimum study-count requirement; for setup outcomes, heterogeneity in error definitions and image-verification workflows left too few studies within comparable system subgroups. Effect magnitudes differed across the analyzable system strata: the largest reduction in Heart Dmean was observed in the OSMS subgroup, whereas the largest reduction in ipsilateral lung V20 was observed in the Catalyst/C-RAD subgroup, followed by OSMS. These are between-study exploratory comparisons intended to describe performance differences among SGRT systems in analyzable outcomes. SGRT =

surface-guided radiotherapy; OSMS = optical surface monitoring system; LAD = left anterior descending coronary artery; MD = mean difference; CI = confidence interval.
